# Sphingolipid Modulation Activates Proteostasis Programs to Govern Human Hematopoietic Stem Cell Self-Renewal

**DOI:** 10.1016/j.stem.2019.09.008

**Published:** 2019-11-07

**Authors:** Stephanie Z. Xie, Laura Garcia-Prat, Veronique Voisin, Robin Ferrari, Olga I. Gan, Elvin Wagenblast, Kerstin B. Kaufmann, Andy G.X. Zeng, Shin-ichiro Takayanagi, Ishita Patel, Esther K. Lee, Joseph Jargstorf, Gareth Holmes, Guy Romm, Kristele Pan, Michelle Shoong, Aditi Vedi, Chiara Luberto, Mark D. Minden, Gary D. Bader, Elisa Laurenti, John E. Dick

**Affiliations:** 1 Princess Margaret Cancer Centre, University Health Network, Toronto, ON M5G0A3, Canada; 2 The Donnelly Centre, University of Toronto, Toronto, ON M5S3E1, Canada; 3 Department of Molecular Genetics, University of Toronto, Toronto, ON M5S1A8, Canada; 4 R&D Division, Kyowa Kirin Co., Ltd., Tokyo 194-8533, Japan; 5 Wellcome-Medical Research Council Cambridge Stem Cell Institute, Department of Haematology, University of Cambridge, Cambridge, UK; 6 Department of Physiology and Biophysics, Stony Brook University School of Medicine, Stony Brook, NY 11794, USA; 7 Division of Medical Oncology and Hematology, Department of Medicine, University Health Network, Toronto, ON, Canada; 8 Department of Medicine, University of Toronto, Toronto, ON, Canada; 9 Department of Medical Biophysics, University of Toronto, Toronto, ON, Canada

**Keywords:** hematopoietic stem cell, umbilical cord blood, autophagy, unfolded protein response, sphingolipid metabolism, fenretinide, DEGS1, lipidomics, UM171, StemRegenin-1

## Abstract

Cellular stress responses serve as crucial decision points balancing persistence or culling of hematopoietic stem cells (HSCs) for lifelong blood production. Although strong stressors cull HSCs, the linkage between stress programs and self-renewal properties that underlie human HSC maintenance remains unknown, particularly at quiescence exit when HSCs must also dynamically shift metabolic state. Here, we demonstrate distinct wiring of the sphingolipidome across the human hematopoietic hierarchy and find that genetic or pharmacologic modulation of the sphingolipid enzyme DEGS1 regulates lineage differentiation. Inhibition of DEGS1 in hematopoietic stem and progenitor cells during the transition from quiescence to cellular activation with *N*-(4-hydroxyphenyl) retinamide activates coordinated stress pathways that coalesce on endoplasmic reticulum stress and autophagy programs to maintain immunophenotypic and functional HSCs. Thus, our work identifies a linkage between sphingolipid metabolism, proteostatic quality control systems, and HSC self-renewal and provides therapeutic targets for improving HSC-based cellular therapeutics.

## Highlights

• Sphingolipid composition is diverse across the human hematopoietic hierarchy
• DEGS1 is a sphingolipid enzyme required for hematopoietic stem cell (HSC) function
• Modulating DEGS1 function activates autophagy and the unfolded protein response
• Variations in sphingolipid homeostasis serve to regulate HSC fate

## Graphical Abstract

**Figure:**
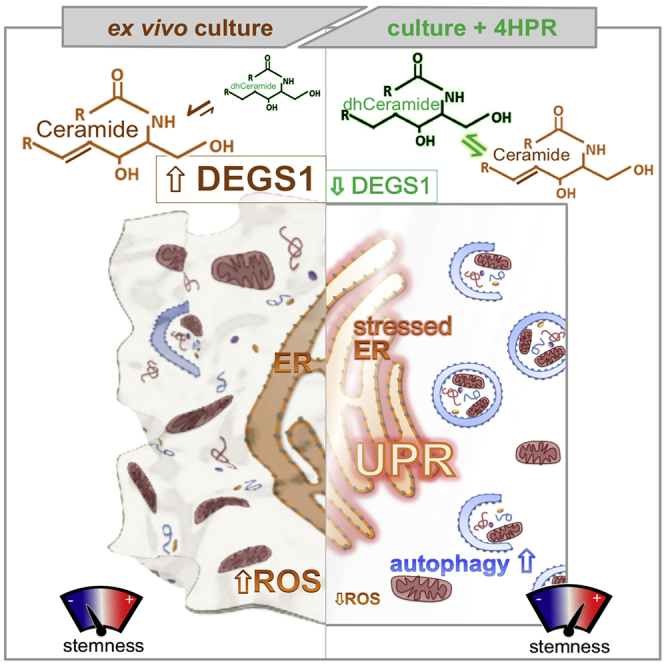

## Teaser

Lipid metabolism is distinctly regulated in human hematopoietic stem cells (HSCs) versus progenitors. Xie et al. profiled the sphingolipidome in human cord blood and show that modulating sphingolipids during the transition from quiescence to cellular activation in *ex vivo* culture induced proteostatic cellular stress programs to maintain HSC self-renewal.

## Introduction

Humans have an enormous demand (∼10^e12^ cells daily) for hematopoietic output. Meeting this need over a lifetime raises an inherent risk for malignancy due to DNA replication errors and potential metabolic and cellular stressors that cause cellular damage (Doulatov et al., 2012, Kohli and Passegué, 2014). Yet hematologic malignancies remain relatively rare. The hierarchical organization of blood is thought to mitigate the risk of transformation. All mature lineages descend from a pool of rare and largely quiescent long-term hematopoietic stem cells (LT-HSCs) that in turn generate highly proliferative but more short-lived populations, including short-term HSCs (ST-HSCs) and committed progenitors. Only LT-HSCs are able to regenerate the entire blood system upon transplantation and maintain long-term output due to their unique capacity for self-renewal. Whereas dormancy in LT-HSCs minimizes the possibility of detrimental mutations passing to all progeny, damage arising within the rapidly proliferating downstream progenitors is ultimately purged upon terminal differentiation (Milyavsky et al., 2010, Mohrin et al., 2010). However, recent studies have found that dormancy is not the only feature that uniquely protects LT-HSCs. Compared to downstream progenitors, stressed or damaged LT-HSCs respond by activating pathways that result in culling rather than pathways that serve to repair or resolve the damage, as is typical for progenitors and most mammalian cell types (Milyavsky et al., 2010, Mohrin et al., 2010). How this unique LT-HSC fate choice is made is poorly understood, particularly upon quiescence exit when LT-HSCs must also dynamically shift metabolic state. The unfolded protein response (UPR) and macroautophagy, hereafter referred to as autophagy, have been identified as critical mediators for HSC stress responses (Ho et al., 2017, van Galen et al., 2014, Warr et al., 2013). More broadly, these new findings revealed an unexplored layer of metabolic and organelle biology that influences LT-HSC regulation of self-renewal, quiescence, proliferation, and lineage commitment that extends beyond the traditional focus on transcription factor networks governing fate decisions (Wilkinson and Göttgens, 2013).

The energy and macromolecule requirements of HSCs are distinct from progenitors, and genetic ablation of key metabolic regulators in mouse models often lead to HSC exhaustion; as such, stringent control of cellular metabolism is fundamental for HSC function (Gan et al., 2010, Gurumurthy et al., 2010, Ito and Suda, 2014, Nakada et al., 2010, Simsek et al., 2010, Takubo et al., 2013, Wang et al., 2014). Recent data suggest that HSCs contain integrated cellular networks coordinating proteostasis with dynamic biosynthetic and metabolic states to govern stem cell fate decisions, especially in the transition from dormancy to cellular activation (Gurumurthy et al., 2010, Mohrin et al., 2015, Warr et al., 2013). Presumably, quality control mechanisms ensure HSC organelle and proteostatic health upon damage incurred from cell cycle entry and other basal metabolic stressors, such as reactive oxygen species (ROS) (García-Prat et al., 2017). However, metabolic as well as translation initiation heterogeneity cannot be attributed entirely to cellular identity differences between LT-HSCs and committed progenitors; dormant HSCs exhibit lower biosynthetic activity and protein synthesis than active HSCs, suggesting HSC metabolic requirements are highly adaptive to cellular state (Cabezas-Wallscheid et al., 2017, Signer et al., 2014). Additionally, the dysregulation of quality control mechanisms (e.g., autophagy) that occur upon HSC aging results in metabolic changes, suggesting that HSC fate is intimately entangled with metabolic and proteostatic regulation (Ho et al., 2017, Kohli and Passegué, 2014). Enhanced endoplasmic reticulum (ER) function in human HSCs with enforced expression of the chaperone ERDJ4/DNAJB9 confers protection against the ER stress that is induced upon xenotransplantation, thereby preserving self-renewing HSCs (van Galen et al., 2014). Moreover, we recently showed higher levels of activating transcription factor 4 (ATF4) and components of the pro-survival integrated stress response (ISR) in human HSCs are cytoprotective during homeostasis, despite their lowered threshold for culling with strong stress stimuli (van Galen et al., 2018). Thus, a complex picture emerges suggesting the nature of the stress stimulus is important to fine-tune quality control responses for HSC persistence or culling. However, these studies raise a number of key questions: is there a single stimulus that activates both autophagy and the UPR to confer cytoprotective functions to HSCs; is there a coordinated cellular stress response upon stress induction; and how do proteostasis programs impact HSC self-renewal?

Proper management of lipid composition is integral for maintaining cellular membrane dynamics for cell division and signaling in cell lines (Atilla-Gokcumen et al., 2014, Köberlin et al., 2015). Although lipid homeostasis is critical for cellular and organismal health, the exploration of lipid metabolism in HSC function is limited (Hotamisligil, 2017, Ito et al., 2012, Ito et al., 2016). Although lipostatic stress as well as proteostatic stress is known to converge on the activation of the UPR for pro-survival in human cells, and autophagy is a crucial element for maintaining lipid homeostasis in mice (Singh et al., 2009, Thibault et al., 2012), there is a critical lack of understanding on the impact of altering lipid composition to HSC function beyond the connection of fatty acid metabolism and mitochondrial function (Ito et al., 2016). Ceramide (Cer), the central component of sphingolipids (SpLs), has been proposed to be part of a lipid biostat that regulates cellular stress and activates stress responses (Hannun, 1996, Hannun and Obeid, 2018). DEGS1 (delta 4-desaturase, Sphingolipid 1, or DES1) is an ER-membrane-spanning protein and the final enzyme in *de novo* SpL synthesis, which converts dihydroceramide (dhCer) to Cer; both genetic ablation and inhibition with the synthetic retinoid fenretinide/*N*-(4-hydroxyphenyl) retinamide (4HPR) are sufficient to activate autophagy in mouse cells or human cell lines (Siddique et al., 2013, Siddique et al., 2015). The potent bioactive lipid sphingosine-1-phosphate (S1P) is generated from metabolism of Cer and exerts pleiotrophic signaling roles in proliferation, survival, and migration of immune cells and HSCs, as well as regulation of lymphocyte lineage commitment and hematopoietic stem and progenitor cell (HSPC) function (Blaho et al., 2015, Golan et al., 2012, Juarez et al., 2012, Massberg et al., 2007, Schwab et al., 2005). Although S1P signaling regulates mouse hematopoiesis, whether other SpLs are important to HSC function is unknown.

Here, we uncovered a transcriptional signature of genes governing SpL synthesis that distinguishes human HSCs and committed progenitors. This distinct SpL wiring in the human hematopoietic hierarchy was confirmed with sphingolipidomic analysis of sorted mature and HSPC populations. When human HSCs are placed in *ex vivo* conditions thought to promote cord blood (CB) HSC activation and expansion, they actually lose HSC function due to impaired proteostatic programs. By contrast, inhibition of DEGS1 in human HSCs with 4HPR treatment before quiescence exit in *ex vivo* culture induced a coordinated response of proteostatic cellular stress programs, including autophagy to maintain HSC self-renewal. Despite *ex vivo* culture, HSCs following SpL modulation functionally show higher self-renewal relative to cultured cells without treatment pointing to a linkage between SpLs, proteostatic quality control programs, and HSC self-renewal in the transition from quiescence to cellular activation.

## Results

### DEGS1 Influences SpL Composition in the Human Hematopoietic Hierarchy and Is Functionally Required for HSC Repopulation

**Figure 1:**
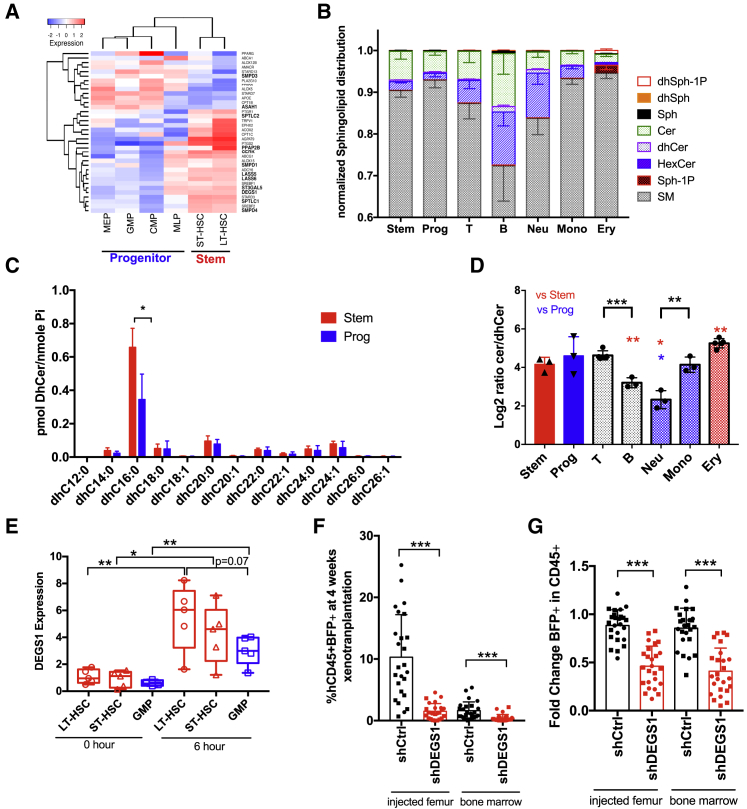
DEGS1 Contributes to the Distinct Wiring of Sphingolipid Synthesis in the Human Hematopoietic Hierarchy and Is Functionally Required *In Vivo* (A) Heatmap of mRNA expression for 36 lipid genes that are significantly differentially expressed (FDR < 0.05 and fold change > 1.5) between LT-HSCs and megakaryocyte erythroid progenitor (MEP)/GMP/common myeloid progenitor (CMP)/multi-lymphoid progenitor (MLP) in the dataset from Laurenti et al. (2013). SpL genes are in bold. (B) SpL distribution in the indicated CB populations (n = 3–5). See Figure S1 for direct comparisons of each SpL. (C) Normalized dhCer profiles for stem and progenitor cells with the indicated fatty-acyl chain (n = 3). (D) Log2 ratio of Cer to dhCer %. Significance to stem cells is in red and to progenitors is in blue. (E) qRT-PCR of DEGS1 at 0 h and 6 h in culture from CB subpopulations. (F) Human engraftment (hCD45^+^BFP^+^) at 4 weeks xenotransplantation for shCtrl or shDEGS1 marked by BFP (5 biological replicates; n = 5 mice per replicate; see Figures S1J–S1M for transduction input, hCD45 chimerism, and BFP%). (G) Fold change of BFP marked transduced cells relative to input in human CD45^+^ cells. Unpaired t test; ^∗^p < 0.05; ^∗∗^p < 0.01; ^∗∗∗^p < 0.001.

We undertook transcriptome analysis of highly resolved subpopulations of the human hematopoietic hierarchy and found that lipid signaling and metabolism genes involved in SpLs are differentially expressed (false discovery rate [FDR] < 0.05; fold change [FC] > 1.5) in LT-HSCs and ST-HSCs (as defined in Laurenti et al., 2015, Notta et al., 2011, Notta et al., 2016) compared to committed progenitors (Figures 1A and S1A). Previous lipid measurements of mammalian cells indicated that SpLs contribute only ∼10% of the cellular lipidome, mostly represented by structural sphingomyelins (SMs) and glycosphingolipids (van Meer and de Kroon, 2011). Overlaying the differentially expressed SpL genes (Figure 1A) onto the metabolic pathway (Hannun and Obeid, 2018) showed many of the SpL genes highly expressed in HSCs centered around those involved in the synthesis of the low abundant bioactive dhCer and Cer species (Figure S1A). To assess whether there is distinct SpL biosynthesis across the cell types comprising the human hematopoietic hierarchy, especially at the level of these less abundant SpLs, we isolated CD34^+^CD38^−^ stem-enriched (stem) and CD34^+^CD38^+^ progenitor-enriched (progenitor) cells and 5 mature blood lineages (B and T lymphocytes, monocytes, neutrophils, and erythrocytes) from CB by flow cytometry. These populations were subjected to Cer, dhCer, sphingosine, S1P, dhSph, dhS1P, hexosylceramides (HexCer) (Cer containing glucose or galactose), and SM measurement using liquid chromatography mass spectrometry (LC-MS) (Figures 1B and S1B–S1H). SMs were the most abundant SpLs in our analysis (Figures 1B and S1H; 72%–94%), consistent with previous lipidome profiling in mammalian cells (van Meer and de Kroon, 2011). Importantly, our profiling identified the accumulation of S1P specifically in erythrocytes (Figure S1E), confirming this lineage-specific association and the robustness of our sphingolipidome profiling (Dahm et al., 2006). We found no significant differences in SpL content between stem and progenitor cells except in the amount of dhCer carrying the C16:0 fatty acid, providing evidence for differential wiring of *de novo* SpL synthesis at the lipid level in HSPCs (Figure 1C). By contrast, the mature lineages showed significant differences from stem and/or progenitor cells (Figures S1C–S1H). Importantly, we saw that B cells, neutrophils, and erythrocytes were significantly different in their ratio of Cer/dhCer from stem cells (Figure 1D). In contrast, T cells and monocytes did not differ in the Cer/dhCer ratio, raising the question of whether Cer homeostasis regulates HPSC fate and lineage commitment decisions. DEGS1 expression levels are significantly increased in LT-HSCs, ST-HSCs, and granulocyte-monocyte progenitors (GMPs) following 6 h of cytokine stimulation, suggesting increasing *de novo* SpL-generated Cer pools may be an early event in the transition from quiescence to cellular activation (Figure 1E). To determine whether alterations in the Cer/dhCer ratio were functionally relevant in HSPC, we modulated their ratio through DEGS1 perturbation and asked whether DEGS1 was required for *in vivo* repopulation. A lentiviral knockdown (KD) construct to DEGS1 was generated that decreased DEGS1 gene expression to 37% of shControl (shCtrl) in a cell line model (Figure S1I). CB stem cells were transduced *in vitro* with either shCtrl or shDEGS1 vectors co-expressing blue fluorescent protein (BFP) and transplanted into mice. At 4 weeks, we found DEGS1 KD significantly decreased human CD45^+^ BFP^+^ chimerism by 2-fold relative to BFP^+^ input and resulted in lineage skewing with an increase in myeloid cells at the expense of B lymphoid cells (Figures 1F, 1G, and S1J–S1O). In summary, we have uncovered considerable diversity in SpL composition across the human hematopoietic hierarchy and found that human HSPCs require DEGS1 *in vivo*.

### Sphingolipid Modulation with the DEGS1 Inhibitor 4HPR Alters HSC Function and Lineage Balance *In Vitro*

**Figure 2:**
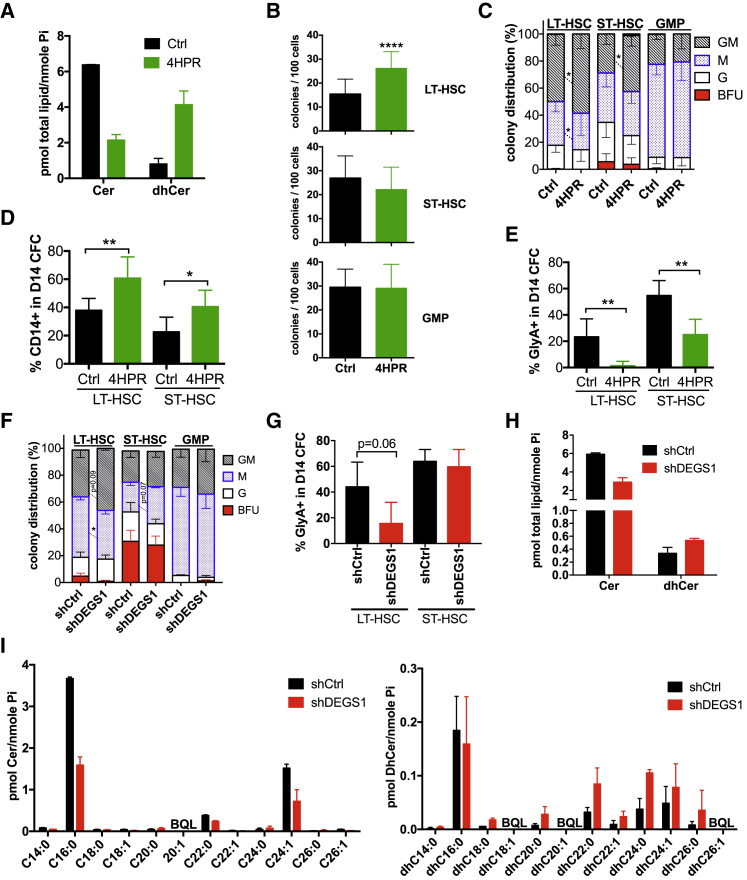
Sphingolipid Modulation of DEGS1 Alters HSC Function and Lineage Balance *In Vitro* (A) Total Cer and dhCer levels in lin^−^ CB progeny cultured for 8 days with control or 4HPR (n = 2). (B and C) LT-HSC, ST-HSC, or GMP CFC assays in the presence of Ctrl or 2 μM 4HPR showing (B) colonies/100 cells and (C) colony distribution (n = 12; CB). (D and E) Flow cytometry for (D) monocytic (CD14^+^) and (E) erythroid (GlyA^+^) markers in live cells from pooled CFC colonies (n = 6; CB). (F) Colony distribution in CFC assays with LT-HSC, ST-HSC, or GMP cells transduced with shCtrl or shDEGS1 (n = 4; CB). (G) Flow cytometry for erythroid cells from pooled CFC colonies in (F). (H and I) hCD45^+^BFP^+^ lacking the CD34^+^CD19^−^ population were isolated from shCtrl or shDEGS1 mice at 4 weeks post-transplant (n = 2 CB) and profiled by LC/MS for (H) total Cer and dhCer levels and (I) normalized Cer and dhCer profiles with the indicated fatty-acyl chain. BQL, below quantitation level. ^∗^p < 0.05; ^∗∗^p < 0.01; ^∗∗∗^p < 0.001.

To determine whether DEGS1 is required as part of the proper transition from quiescence to cellular activation and/or self-renewal maintenance in human HSCs, its function must be inhibited at the start of quiescence exit. As lentiviral transduction of CD34^+^ cells requires some period of cytokine pre-stimulation to induce cellular activation (Amirache et al., 2014), we used the irreversible DEGS1 inhibitor 4HPR at 2 μM (Rahmaniyan et al., 2011, Siddique et al., 2015) at the start of cytokine activation. This dose caused 50% maximal reduction in total cells of lineage-depleted CB (lin-CB) cells after 3 days in culture (Figure S2A). 4HPR treatment for 8 days altered SpL composition, resulting in decreased Cer and increased dhCer compared to controls (Figures 2A, S2B, and S4A–S4D). Bromodeoxyuridine (BrdU) incorporation in LT-HSCs, ST-HSCs, and GMPs was decreased by 4HPR treatment at day 3, suggesting modulating Spls in HSPCs decreases proliferation *in vitro* (Figure S2C). Nonetheless, 4HPR treatment still permits quiescence exit and does not significantly alter cell viability (Figures S2D and S2E). Similarly, there was no difference in cell cycle distribution between shCtrl and shDEGS1 in LT-HSCs or in GMPs isolated from mice at 4 weeks transplantation, suggesting DEGS1 KD does not lock cells into quiescence *in vivo* (Figure S2F). Next, we utilized the colony-forming cell assay (CFC) to assess *in vitro* progenitor function to determine whether DEGS1 inhibition alters functional output from HSPC subpopulations. 4HPR treatment significantly increased clonogenic output specifically from LT-HSCs (50% over control), but not from ST-HSCs or GMPs (Figure 2B). Treatment of LT-HSCs and ST-HSCs with 4HPR led to an increased proportion of granulocyte-macrophage (GM) and monocyte (M) colonies (Figure 2C). Flow cytometry confirmed the morphologic CFC assessment that 4HPR enhanced myeloid output at the expense of erythroid (Figures 2D and 2E). As 4HPR resembles all-trans retinoic acid (ATRA) and may have additional effects independent of DEGS1, CFC assays were performed on LT-HSCs, ST-HSCs, and GMPs treated with ATRA but did not mimic 4HPR treatment (Figure S2G). Although 4HPR effects appeared independent of the retinoid pathway, other potential off-target effects on HSC function were possible. Thus, we turned to a genetic approach with DEGS1 KD and transduced LT-HSCs, ST-HSCs, and GMP with shCtrl or shDEGS1 vectors and sorted BFP^+^ transduced cells for CFC assays. We found that shDEGS1 LT-HSCs showed similar but more modest changes in colony distribution and loss of erythroid output (Figures 2F and 2G). The more modest effects of DEGS1 KD may be reflective of the more modest but measurable changes in Cer and dhCer levels compared to control KD in cells isolated from xenografts (Figures 2H, 2I, and S2H). Thus, DEGS1 modulation pharmacologically or via KD alters lineage balance *in vitro* and 4HPR modulation of SpL homeostasis selectively enhances clonogenic efficiency only in LT-HSCs.

### *Ex Vivo* Treatment with 4HPR Maintains HSC Function following Xenotransplantation

**Figure 3:**
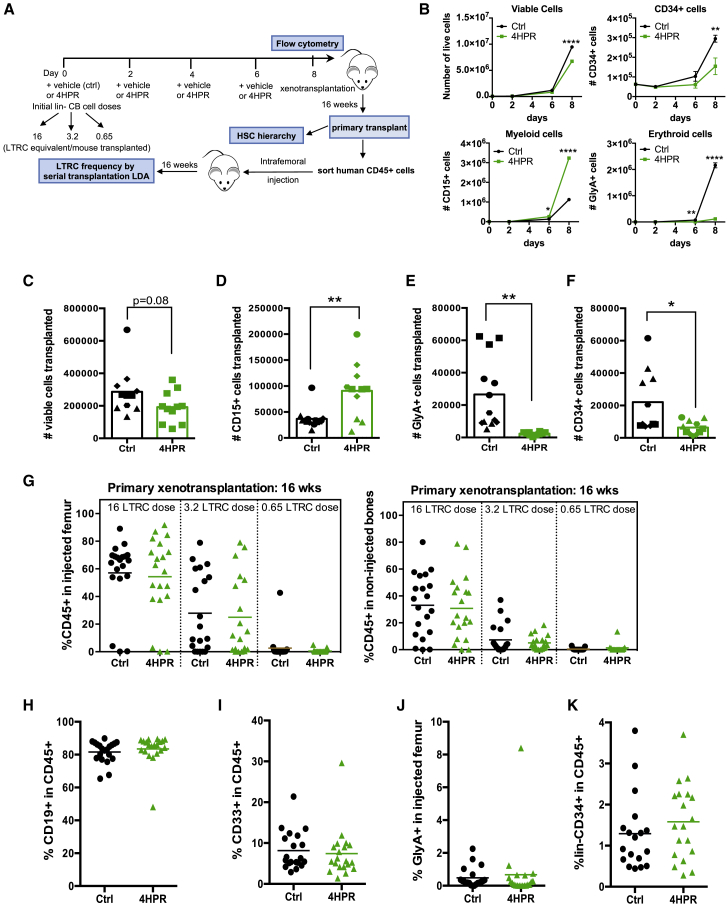
*Ex Vivo* Treatment with 4HPR Maintains HSC Function following Xenotransplantation (A) Experimental scheme for *ex vivo* culture of lin^−^ CB followed by xenotransplantation with vehicle control or 4HPR. (B) Total cell counts at 8 days culture for the 16-LTRC dose of a representative experiment (n = 3; CB cultured in triplicate). (C–F) The number of (C) viable cells, (D) CD15^+^ myeloid cells, (E) GlyA^+^ erythroid cells, and (F) CD34^+^ cells injected/mouse were calculated for the 16-LTRC dose by flow cytometry analysis for control or 4HPR treatment prior to xenotransplantation (n = 5 CB pools, 4 in technical triplicate, marked with different symbols). (G) Human CD45^+^ engraftment at 16 weeks post-transplant in injected femurs and non-injected bones (n = 4 CB pools, 5 mice/drug treatment for each CB pool). (H–K) Lineage analysis of mice engrafted with control or 4HPR-treated cells from Figure 2G for (H) B lymphoid, (I) myeloid, (J) erythroid, and (K) CD34^+^ lacking CD33 or CD19 markers at the 16-LTRC dose. ^∗^p < 0.05; ^∗∗^p < 0.01; ^∗∗∗^p < 0.001.

To directly test whether 4HPR modulation of SpL impacts LT-HSC function, we utilized the xenograft assay to selectively readout self-renewal capacity from culture-generated progeny with HSC function. Our approach to test 4HPR was guided by *ex vivo* expansion methods (Kiernan et al., 2017) that are used clinically to expand CB-derived HSPCs, as single CB units do not contain enough HSCs to enable HSC transplantation (HSCT) into adult recipients in the clinic. Thus, we added 4HPR into an expansion scheme with lin^−^ CB cells containing LT-HSCs, ST-HSCs, and committed progenitors that was similar to recently developed state-of-the-art methods for CB transplantation (Fares et al., 2014). Three initial cell concentrations corresponding to high (16), medium (3.2), and limiting (0.65) long-term repopulating cell (LTRC) doses were cultured *ex vivo* for 8 days mimicking fed-batch conditions (Csaszar et al., 2012) and then their progeny were analyzed by flow cytometry and assessed for engraftment potential by xenotransplantation (Figure 3A; see STAR Methods). 4HPR-treated cultures had fewer CD34^+^ cells, enhanced myeloid differentiation, and decreased erythroid differentiation (Figures 3B–3F). Despite fewer CD34^+^ cells at transplantation, human CD45 chimerism for control and 4HPR-treated progeny were similar in all cell doses following xenotransplantation (Figure 3G). Similarly, lineage distribution for B lymphoid, myeloid, and erythroid lineages (Figures 3H–3J) and the percentage of lin^−^ CD34^+^ cells (Figure 3K) from the 16-LTRC dose was comparable for control and 4HPR-treated groups. Flow cytometry analysis of the HSC hierarchy in individual mice confirmed all HSPC subpopulations analyzed exhibited similar experimental variation between mice transplanted with control and 4HPR-treated cells (Figures S3A–S3I). These data indicate that, despite reduced CD34^+^ cell output, the quality and quantity of functionally defined repopulating HSCs from 4HPR-cultured cells at 16 weeks xenotransplantation was similar to controls where far higher numbers of CD34^+^ cells were transplanted. Moreover, lineage differentiation effects resulting from *ex vivo* modulation of SpLs in HPSC by 4HPR are transient and reversible following xenotransplantation because the number of HSPCs regenerated in the mice was equivalent to controls. In contrast, when DEGS1 is genetically modulated via KD, we observed a severe decrease (4.5-fold decrease over shCtrl; Figure S3J) in the number of CD34^+^ at 4 weeks *in vivo*. Hence, reducing DEGS1 activity transiently or persistently restrains the generation of CD34^+^ progeny.

### Sphingolipid Modulation Restricts Expansion of Committed Progenitors during *Ex Vivo* Culture to Enhance HSC Self-Renewal

**Figure 4:**
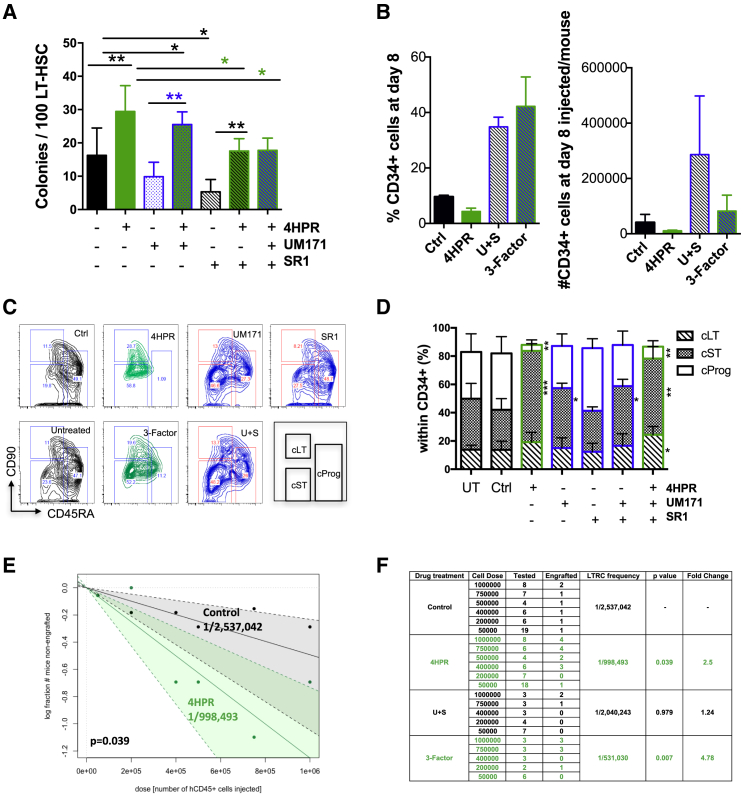
Sphingolipid Modulation Restricts Expansion of Committed Progenitors during *Ex Vivo* Culture to Enhance HSC Self-Renewal (A) Number of colonies for LT-HSC CFC assays with control (−) or the indicated combination of 4HPR, UM171, or SR1 at 10 days, unpaired t test relative to control (Ctrl, black) or to the presence or absence of 4HPR treatment. (B) Flow cytometry for the % and count (no.) of CD34^+^ cells subsequently transplanted per mouse following 8 days culture. (C) Representative flow cytometry plots for CD90 and CD45RA within the CD34^+^ fraction of lin^−^ CB progeny at day 8 with the indicated treatments. (D) Distribution of cultured CD34^+^CD90^+^CD45RA^−^ (cLT), CD34^+^CD90^−^CD45RA^−^ (cST), and CD34^+^CD90^−^CD45RA^−^ (cProg) for the treatments at day 8 following CD34 enrichment (n = 4); unpaired t test relative to control. (E and F) Graph of LTRC frequencies for control and 4HPR (E) and table summarizing results of secondary assays (F) with CD45^+^ cells isolated from 16-LTRC dose mice and transplanted at limiting doses for 16 weeks to calculate LTRC frequencies (5 biological experiments for control and 4HPR; 2 biological experiments for U+S and 3-Factor). Human CD45^+^ marking of >0.1% was considered positive for secondary engraftment. p value was by extreme limiting dilution analysis (ELDA). ^∗^p < 0.05; ^∗∗^p < 0.01; ^∗∗∗^p < 0.001.

To determine whether 4HPR treatment caused a potential dissociation between phenotypic and functional HSPCs during culture, we quantitatively assess the HSC self-renewal capacity of 4HPR-treated CB progeny using serial transplantation approaches. In contrast to previous strategies that focused on the expansion of CD34^+^ cells to improve the clinical utility of CB as a HSCT source, SpL modulation by 4HPR here restrained proliferation and resulted in 3.3-fold fewer CD34^+^ cells relative to control treatment (Figure 3F). Therefore, we investigated in parallel the effects of two known CB CD34^+^ agonists UM171 and SR1 (Fares et al., 2014, Boitano et al., 2010) alone and in combination with 4HPR. First, we assessed the clonogenic activity of LT-HSCs in CFC assays and found that 4HPR significantly increased clonogenic activity in combination with either UM171 or SR1 to levels similar to 4HPR alone (Figure 4A). In contrast, UM171 or SR1 alone did not increase clonogenic activity of LT-HSCs. Next, we cultured 2 independent pools of lin^−^CB with DMSO control, 4HPR, UM171+SR1 (U+S), and UM171+SR1+4HPR (3-Factor) for 8 days and found 4HPR treatment restricted the percentage and number of CD34^+^ cells, and 3-Factor treatment resulted in an enhanced percentage of CD34^+^ cells comparable to U+S treatment (Figure 4B). The total number of cells following 8 days culture was similar between 4HPR and 3-Factor treatment (Figure S4A). We performed LC-MS analysis and found the SpLs measured in 3-Factor progeny phenocopied 4HPR treatment and were similarly perturbed (Figures S4B–S4D). We further analyzed CD34^+^ cells at day 8 culture for cultured LT-HSCs (cLT:CD90^+^CD45RA^−^), ST-HSCs (cST:CD90^−^CD45RA^−^), and cultured committed progenitors (cProg:CD90^−^CD45RA^+^; Figure 4C). 4HPR alone significantly limited the percentage of cProg cells while increasing cST cells in the CD34 compartment and trended to increased cLT cells relative to control; 3-Factor treatment resulted in significant enrichment of both cLT and cST populations (Figures 4C and 4D). The functional identity of these subpopulations was confirmed in an independent experiment by xenotransplantation for 16 weeks; as expected, cProg cells were unable to give rise to human CD45 grafts (Figures S4E–S4G). Hence, these data show 4HPR acts dominantly over UM171 or SR1 to enhance LT-HSC clonogenic ability, and 3-Factor treatment during *ex vivo* culture increases immunophenotypic HSC subsets.

To determine whether 4HPR alone or 3-Factor enhances *in vivo* HSC self-renewal following *ex vivo* treatment, lin^−^ CB progeny cultured with control, 4HPR, U+S, or 3-Factor for 8 days were serially transplanted into NSG mice (Figures 3G and S4H). In line with previous work on UM171 and SR1, U+S or 3-Factor *ex vivo* treatment significantly increased primary engraftment of CB cells (Figure S4H; Fares et al., 2014). Next, human CD45^+^ cells engrafted at 16-LTRC doses were serially transplanted in a limiting dilution assay (LDA) to enumerate functional LTRCs from the primary recipients for 5 independent pools of CB treated with 4HPR; the total period of engraftment was 32 weeks. 4HPR-treated cells showed a significant 2.5-fold increase in LTRC frequency over control upon secondary transplantation (Figures 4E and 4F; p = 0.039). In the 2 experiments where U+S were also assessed, 4HPR alone resulted in 2.1-fold increase in LTRC frequency, similar to the frequency obtained by combining the five experiments (Figures 4E, 4F, S4I, and S4J). However, the LTRC frequency of U+S-treated cells was similar to control. Importantly, 3-Factor-treated cells gave a 4.7-fold augmentation in LTRC frequency over cultured control cells (p = 0.007). Collectively, these transplantation data showed that CB expansion with 4HPR alone and in combination with U+S successfully sustained a pool of functional HSCs during *ex vivo* culture with enhanced *in vivo* HSC self-renewal capacity relative to controls.

### 4HPR Activates a Coordinated Cellular Stress Response, including Autophagy and the UPR/ISR during *Ex Vivo* Culture

**Figure 5:**
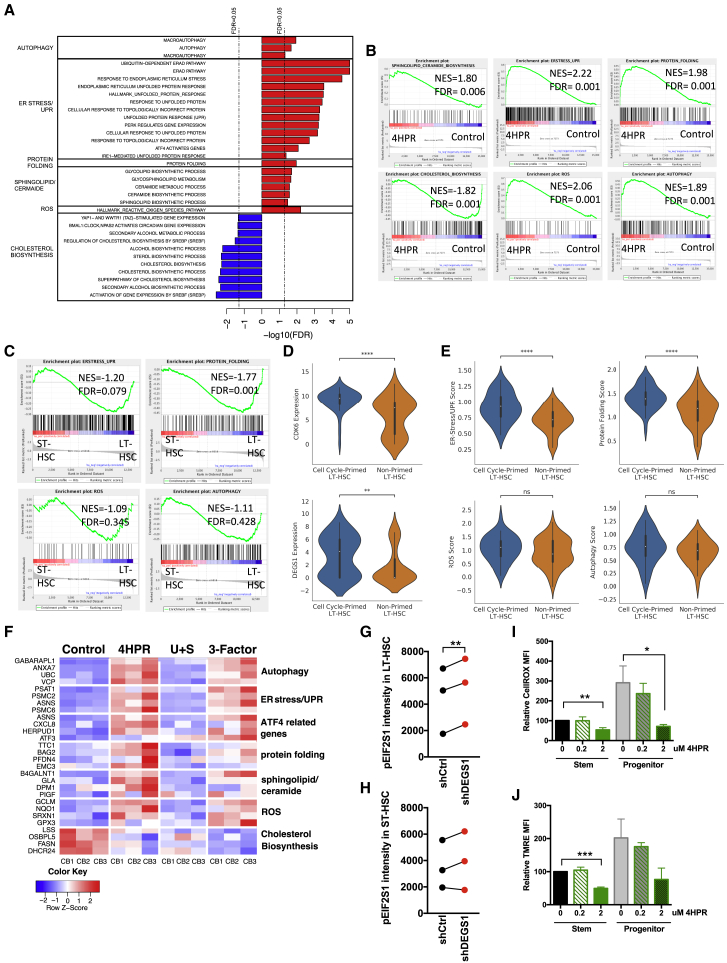
Sphingolipid Modulation with 4HPR Treatment Upregulates Cellular Stress Pathways and Remodels Cellular Metabolism in HSPCs during *Ex Vivo* Culture (A) NES scores for selected pathways that are significantly enriched following 4HPR treatment at day 2. (B) Pathway analysis (GSEA) of SpL/cer, cholesterol biosynthesis ER stress/UPR, protein folding, and ROS pathway modules for 4HPR compared to control. (C) GSEA analysis of autophagy, ER stress/UPR, protein folding, or ROS pathways in uncultured LT-HSCs versus ST-HSCs. (D and E) LT-HSCs from Velten et al. were clustered as cell cycle primed or non-primed as described in STAR Methods, (D) the gene expression of CDK6 (top) and DEGS1 (bottom), and (E) signature scores representing relative expression of pathways in (C) for single LT-HSCs; Wilcoxon rank sum test for (D) and (E); ^∗^p < 0.05; ^∗∗^p < 0.01; ^∗∗∗^p < 0.001; ^∗∗∗∗^p < 0.0001. (F) Heatmap showing gene expression at day 2 of the top 4 genes in gene sets in (A) organized as pathways. We included ATF4-related genes gene sets from the ER stress/UPR pathway. (G and H) Median pEIF2S1 staining intensity quantified from microscopy images for BFP^+^ (G) LT-HSCs and (H) ST-HSCs isolated from 4-week xenografts engrafted with shCtrl or shDEGS1 stem cells for 3 CBs (60 cells/CB, except shDEGS1 LT-HSCs, 13–17 cells/CB). (I and J) Flow cytometry analysis at day 2 post-treatment with indicated concentrations of 4HPR in the progeny of CD34^+^CD38^−^ stem or CD34^+^CD38^+^ progenitor CB cells from 4 CBs for (I) ROS with CellROX and (J) mitochondrial membrane potential with TMRE. Paired t test; ^∗^p < 0.05; ^∗∗^p < 0.01; ^∗∗∗^p < 0.001 in (G)–(J).

To elucidate the biological pathways altered by 4HPR treatment in human HSPCs that contributed to lineage differentiation effects and maintenance of HSC self-renewal during cytokine activation, we performed RNA sequencing (RNA-seq) on lin^−^ CB cells at days 2 and 4 following treatment with control, 4HPR, U+S, and 3-Factor (n = 3). GSEA pathway analysis identified 473 significantly upregulated gene sets and 80 significantly downregulated gene sets (FDR < 0.05) in 4HPR compared to controls at day 2 (Figure S5A; Table S1). Many of the upregulated pathways following 4HPR treatment centered on cell cycle progression, which is consistent with the decreased S phase progression in HSPC subsets we observed following 4HPR treatment (Table S1; Figure S2C). Lipid metabolism pathways were significantly altered with an upregulation of Cer/SpL biosynthesis and a decrease in cholesterol/sterol biosynthetic pathways (Figure 5A), possibly a feedback response due to SpL homeostasis disruption by 4HPR (Köberlin et al., 2015, Singh et al., 2009). Interestingly, a strong cellular stress induction theme was highlighted in the pathway analysis (FDR < 0.05; Figure S5A) following 4HPR treatment, including ER stress/UPR/ATF4, protein folding, ROS, and autophagy. These pathways were grouped into 6 modules, and GSEA showed a significant enrichment of these pathways for 4HPR treatment relative to control (Figures 5A and 5B; shaded gray on Figure S5A; gene lists in Table S2).

We asked whether some of these stress response programs following 4HPR-induced lipostatic stress are potentially intrinsic HSC maintenance mechanisms necessary for cellular activation. When we analyzed ER stress/UPR, protein folding, ROS, and autophagy in uncultured LT-HSCs compared to uncultured GMPs by GSEA, only autophagy was not significantly enriched, suggesting some stress response programs are more activated in progenitors than HSCs at homeostasis (Figure S5B). To distinguish cell cycle priming as opposed to lineage priming differences between LT-HSCs and GMPs, we compared ST-HSCs to LT-HSCs at homeostasis, and we found a significant enrichment of protein folding (p = 0.001) and near significant enrichment of ER stress/UPR (p = 0.079) in LT-HSCs, suggesting these programs may be required for quiescence exit and/or self-renewal (Figure 5C). Additionally, we analyzed published single-cell RNA-seq data of HSCs from bone marrow (Velten et al., 2017). Within CD34^+^CD38^−^CD45RA^−^ cells, we identified two clusters, cell-cycle-primed and non-primed, using a clustering algorithm developed to robustly find distinguishing gene expression features in stem cells (Tarashansky et al., 2019; see STAR Methods). We specifically examined LT-HSCs, which were represented in both clusters, and found that cell-cycle-primed LT-HSCs have higher CD38 surface expression, cell cycle programs, and CDK6 and DEGS1 expression than non-primed LT-HSCs (Figures S5C, S5D, 5D, and 5E). The latter had enrichment for a set of dormancy-associated genes found in label-retaining dormant mouse LT-HSCs (Figure S5E; Cabezas-Wallscheid et al., 2017). We found ER stress/UPR and protein folding enriched in cell-cycle-primed LT-HSCs, suggesting the activation of ER proteostasis programs as well as upregulation of DEGS1 gene expression is an early event in HSC activation.

Next, we asked how the gene expression changes enacted by 4HPR treatment compared to combination treatment with U+S during culture and changed over time. Cells subjected to 3-Factor treatment at day 2 showed negative enrichment for cholesterol/sterol biosynthesis pathways and at day 4 also showed enrichment of ER stress and UPR pathways as observed with 4HPR alone (Table S1). Gene expression changes for the top 4 genes in the selected pathways are similar following 4HPR treatment and 3-Factor treatment, but 3-Factor-treated samples show fewer significantly altered gene sets than with 4HPR treatment alone at both days 2 and 4 (Figures 5F, S5F, and S5G). These data point to activation of autophagy and ROS gene sets as resulting exclusively from 4HPR treatment. Although cytokine treatment induces cell cycle entry but no cell division in LT-HSCs at day 2 (progenitors have divided), most LT-HSCs have completed one cellular division only by day 4 (Figure S2D; Laurenti et al., 2015). Nonetheless, gene expression changes in ER stress/UPR and metabolic gene sets persist from day 2 to day 4 (Table S1; Figures S5F–S5H), suggesting 4HPR treatment is provoking a sustained HSC maintenance response in *ex vivo* culture independently of cell cycle transit following quiescence exit.

We had previously identified a cytoprotective ATF4-ISR program downstream of the UPR in response to metabolic stress in human HSCs (van Galen et al., 2018). Because we retrieved enrichment in the ER stress/UPR functional model for an “ATF4 activated genes” gene set by 4HPR treatment, we asked whether HSC subsets following DEGS1 KD also showed evidence for activation of this UPR/ATF4 program. We quantified phosphorylated eukaryotic translation initiation factor 2α (eIF2α) in LT-HSCs and ST-HSCs from 4-week xenografts engrafted with shCtrl- or shDEGS1-transduced HSPCs by confocal microscopy (Figures 5G and 5H; 3 CB from experiment 2 of Figures S1J–S1M). Upon UPR activation by ER stress, eIF2α is phosphorylated to attenuate eIF2B, resulting in global translation inhibition except for the specific transcriptional programs, e.g., ATF4, licensed by the UPR (Hetz et al., 2013). As LT-HSCs are activated, with 60% in cycle at 4 weeks transplant, the presence of anti-pEIF2S1/phospho-eIF2α staining in shCtrl LT-HSC and ST-HSC reflects activation of an UPR/ISR program (Figures 5G, 5H, S2F, and S5I; Laurenti et al., 2015, van Galen et al., 2018). Importantly, shDEGS1 LT-HSCs have significantly increased pEIF2S1 staining over shCtrl, suggesting LT-HSCs are more sensitive to lipostatic stress than ST-HSCs *in vivo* (Figures 5G, 5H, S5J, and S5K). Thus, these data suggest SpL modulation via pharmacological inhibition *ex vivo* and via lentiviral KD *in vivo* are activating an UPR/ATF4 program during cellular activation to restore homeostasis.

Metabolic dysregulation through increase in oxidative stress and mitochondrial dysfunction are known activation signals for ATF4-ISR programs (Kasai et al., 2019). Pathways related to redox homeostasis, mitochondria gene sets, and Pink-Parkin mediate mitophagy, suggesting 4HPR treatment has activated the UPR/ATF4 to enact metabolic remodeling to restore homeostasis (Table S1). Therefore, we analyzed reactive oxygen species (ROS) and mitochondrial membrane potential following 2 days of 4HPR treatment in the progeny of CB stem and progenitor cells and found a decrease for both parameters following 4HPR treatment as compared to controls (Figures 5I, 5J, S5L, and S5M). Such metabolic reprogramming by 4HPR persists to day 3 when the progeny of HSPCs, including LT-HSCs treated with 4HPR, have exited quiescence and are actively cycling (Figures S5L, S5M, and S2D). In summary, as DEGS1 functions at the ER to convert dhCer to Cer, which is then further processed at the Golgi apparatus to form complex SpLs (Thibault et al., 2012, Zheng et al., 2006), these data are consistent with 4HPR inhibition of DEGS1 as a lipostatic stress stimulus capable of activating coordinated transcriptional stress programs to remodel the cellular metabolism of cultured HSPCs for stress recovery and ultimately preserve functional HSCs.

### Sphingolipid Modulation Specifically Activates Autophagy in Stem and Not Progenitor Cells

**Figure 6:**
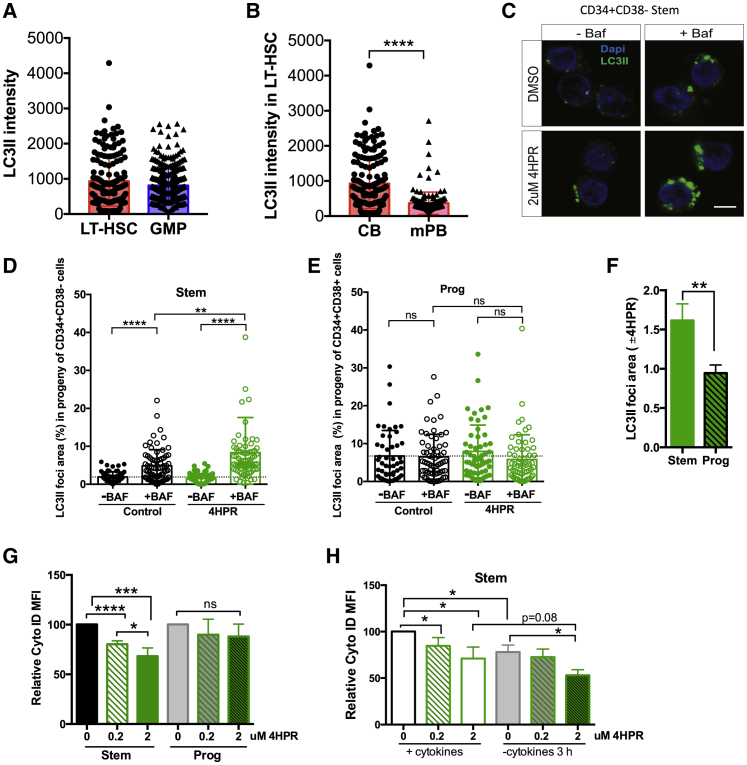
4HPR Activates Autophagy in HSPCs during *Ex Vivo* Culture (A and B) LC3II staining intensity of (A) LT-HSCs (174 cells) and GMPs (312 cells) isolated from CB (n = 3) or (B) mPB (165 cells; n = 3 mPB) LT-HSCs compared to CB LT-HSCs. (C) Representative microscopy images of DAPI (blue) and LC3II staining (green) for (C) CD34^+^CD38^−^ (stem) cells following 2 days of treatment with DMSO control or 2 μM 4HPR and ±BAF. Scale bar is 5 μm. (D and E) LC3II foci area for (D) stem and (E) prog cells from one of three CB. The mean in control stem cells without BAF is shown with a dotted line. (F) Relative LC3II foci in the presence of BAF for stem and Prog populations. (G) Relative Cyto-ID flux for 0 (DMSO), 0.2 μM, and 2 μM 4HPR in stem and prog populations at 2 days post-treatment (n = 4). (H) Relative Cyto-ID MFI measurements for stem-enriched samples to analyze autophagic flux with and without cytokine withdrawal with indicated drug treatments at day 2. ^∗^p < 0.05; ^∗∗^p < 0.01; ^∗∗∗^p < 0.001.

As activating autophagy can promote HSC function (Chen et al., 2009, Luo et al., 2014), we asked whether 4HPR treatment leads to autophagy activation for HSC maintenance during *ex vivo* culture. Gene expression analysis showed SpL modulation by 4HPR upregulated autophagy gene sets in lin^−^ CB. However, autophagy has not previously been characterized in human LT-HSCs in quiescence or upon cellular activation. Thus, we examined basal autophagy in CB LT-HSCs and GMPs with the autophagosome marker LC3II by confocal microscopy and found similar LC3II intensity consistent with murine data (Figures 6A, S6A, and S6B; Ho et al., 2017). To ascertain whether *in vivo* cellular activation disrupts basal autophagy levels in human HSCs, we compared LC3II staining in LT-HSCs isolated from granulocyte-colony-stimulating factor (G-CSF) mobilized peripheral blood (mPB) (Figures 6B and S6C) and found a significant decrease in LC3II intensity in mPB LT-HSCs compared to CB LT-HSCs. Next, we asked whether 4HPR treatment specifically activated autophagy in human CB stem cells and not progenitors, because only murine stem cells appear capable of activating autophagic flux in response to metabolic stress (Warr et al., 2013), with two independent assays: (1) LC3II staining with and without bafilomycin A1 (BAF), an agent that inhibits autophagosome turnover by blocking lysosome acidification (Figures 6C–6F, S6D, and S6E), and (2) flow cytometry with Cyto-ID (Figures 6G, 6H, S6G, and S6H). LC3II staining showed both control and 4HPR-treated stem and progenitor cells at day 2 exhibit basal autophagy in culture (Figures 6D, 6E, and S6D). However, only stem, but not progenitor, cells showed autophagy activation by an increase in LC3II foci area in the presence of 4HPR and BAF (Figures 6D–6F). Similarly, Cyto-ID staining with control or 4HPR-treated cells (0.2 μM or 2 μM) showed that 4HPR significantly amplified autophagic flux only in stem, but not in progenitor, cells (Figure 6G). We confirmed cytokine withdrawal activates autophagy (as measured by Cyto-ID) in human stem, but not progenitor, cells like in mouse (Figures 6H, S6F, and S6G), which is further enhanced by 4HPR (Figure S6H). Collectively, these data indicate 4HPR activated autophagy exclusively in stem cells during *ex vivo* culture.

### Sphingolipid Modulation by 4HPR Activates a Coordinated Proteostatic Pro-survival Response

**Figure 7:**
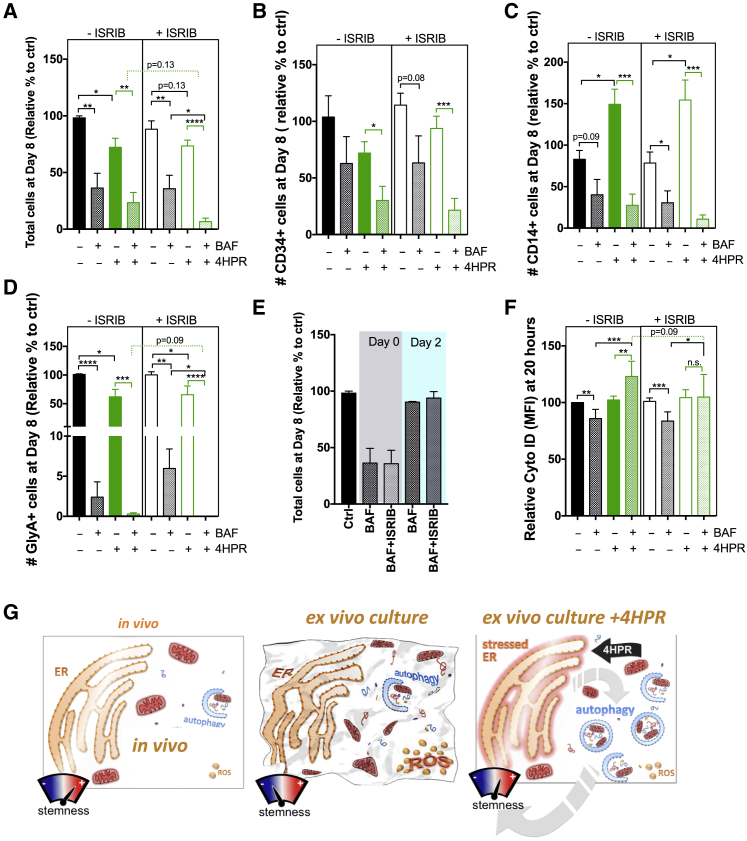
Sphingolipid Modulation by 4HPR Activates a Coordinated Proteostatic Pro-survival Response (A–D) Flow cytometry analysis at day 8 culture following autophagy inhibition with BAF and/or ISR inhibition with ISRIB in lin^−^ CB (n = 3, in duplicate) beginning at day 0 for control or 4HPR-treated cells for number of (A) live cells, (B) CD34^+^ cells, (C) CD14^+^ cells, and (D) GlyA^+^ cells; represented as relative % to control; mean ± SEM. (E) Total number of cells at day 8 after BAF was added either starting at day 0 or day 2. (F) Autophagic flux at 20 h with indicated drugs was assayed with Cyto-ID MFI relative to control (2 CB, in triplicate). (G) Model for how 4HPR maintains stemness during *ex vivo* culture. ^∗^p < 0.05; ^∗∗^p < 0.01; ^∗∗∗^p < 0.001.

To mechanistically determine whether HSPCs require both autophagy and the UPR/ISR for survival in response to SpL modulation with 4HPR in culture, we pharmacologically inhibited autophagy with BAF alone or in combination with integrated stress response inhibitor (ISRIB) in lin^−^ CB. ISRIB is a potent ISR inhibitor by maintaining activity of eIF2B and retaining protein translation despite EIF2α phosphorylation (Hetz et al., 2013). At day 8, the percentage of total number of cells relative to control, CD34^+^ cells, CD14^+^ myeloid cells, and GlyA^+^ erythroid cells were quantified by flow cytometry analysis and compared to vehicle (Figures 7A–7D). ISRIB appeared to have no significant effect on CB lin^−^ cells treated with or without 4HPR by the parameters we measured but significantly decreased proliferation when autophagy was also inhibited. Although BAF treatment was equally potent in restraining proliferation and differentiation particularly to the erythroid lineage in both control and 4HPR-treated CB cells, as previously shown in embryonic stem cells and K562 cells (Zyryanova et al., 2018), the addition of ISRIB with BAF significantly limited the number of viable cells and erythroid cells with 4HPR treatment relative to BAF+ISRIB alone and trended to significance in cells treated with 4HPR+BAF relative to 4HPR+BAF+ISRIB (Figures 7A and 7D). However, BAF treatment in lin^−^ CB cells was context dependent as addition of BAF at day 2, perhaps when culture-induced stress was lower, had minimal effect on cell growth except for erythroid differentiation (Figures 7E and S7B–S7F; Laurenti et al., 2015). Finally, lin^−^ CB cells were treated with the same combination of drugs as in Figure 7A, beginning at quiescence for 20 h, and then Cyto-ID was used to assess whether autophagic flux activation requires coordinate activation of the UPR/ISR (Figure 7F). As these cells are a mix of stem and progenitor cells, no difference between control and 4HPR-treated cells was observed. However, upon BAF addition to accumulate autolysosomes, a loss of flux demonstrated by an increase in Cyto-ID mean fluorescence intensity (MFI) was observed in 4HPR+BAF cells relative to 4HPR cells alone or control + BAF-treated cells (Figure 7F). At this time point, BAF treatment successfully blocked lysosome acidification as measured by flow cytometry with LysoTracker (Figure S7G). Importantly, the accumulation of autolysosomes seen between cells treated with 4HPR+ISRIB and cells treated with 4HPR+ISRIB+BAF was abolished, suggesting some aspect of the ISR may be required to activate autophagic flux with 4HPR treatment (Figure 7F). In summary, these data, along with the serial transplantation assays, support a model (Figure 7G) where SpL modulation by 4HPR in *ex vivo* culture activates a coordinated stress response, including autophagy and ER stress programs, to restore cellular homeostasis and maintain stemness in 4HPR-treated cells during the transition from quiescence to cellular activation.

## Discussion

Here, we provide direct evidence that sphingolipid composition regulates HSC self-renewal and lineage commitment. Although short-term culture normally results in marked CD34^+^ cell expansion at the expense of LT-HSCs, our data showed that modulation of SpL metabolism during culture activates cellular stress proteostasis programs that collectively promote the proper metabolic transition from quiescence to cellular activation, resulting in LT-HSC maintenance. Four findings emerge from our work: (1) the sphingolipidome has distinct composition within the various mature, progenitor, and HSC subsets that comprise the human hematopoietic hierarchy; (2) KD or pharmacological dysregulation of DEGS1 is sufficient to alter the lineage determination of HSPCs; (3) dysregulation of Cer and dhCer homeostasis serves as a signal to activate cellular stress proteostasis programs, including the UPR and autophagy in human HSPCs; and (4) transient SpL modulation by 4HPR during *ex vivo* expansion culture results in the maintenance of functionally defined LT-HSCs that possess serial repopulating ability following xenotransplantation.

Membranes are increasingly being recognized as playing central roles in cellular homeostasis, and Cer pools have a special role in cellular lipid metabolism (Contreras et al., 2010, Hannun and Obeid, 2018). Here, we establish SpL homeostasis as another aspect of lipid metabolism beyond fatty acid oxidation (Ito et al., 2016) that is pivotal to physiological human hematopoiesis. Cer and the complex SpLs are essential components of multiple cellular membranes, including the ER, Golgi, mitochondria, lysosomes, autophagosomes, and at the plasma membrane (Hannun and Obeid, 2018). Cer is generated either via *de novo* synthesis or from the salvage pathway through catabolic membrane recycling. We found that LT-HSCs and ST-HSCs favor increased expression of *de novo* synthesis pathway genes compared to more activated progenitors. We undertook sphingolipidome mapping of the human hematopoietic hierarchy and found distinct composition of sphingolipid species between five mature blood lineages and HSPCs, particularly around the interface of cer to dhCer homeostasis. By inhibiting the *de novo* SpL metabolic enzyme DEGS1 that adds a double bond to dhCer to form Cer, we modulated these *de novo* Cer pools and found that cellular stress programs were initiated. Our findings point to SpL remodeling as an early event in cellular activation of quiescent HSPCs.

Studies from the obesity and aging fields have identified mild induction of ER stress/UPR and autophagy as improving healthspan and lifespan, possibly through effects on proteostasis and organelle homeostasis within stem cell populations (García-Prat et al., 2017, Hotamisligil, 2017). Recently, we reported that ATF4 activates the ISR upon amino acid deprivation to increase HSC survival (van Galen et al., 2018). Moreover, physiological aging of HSCs appears to result in part through suppression of autophagy programs that maintain metabolic homeostasis (Ho et al., 2017, Mohrin et al., 2015). Although a number of ATF4 target genes are known to be involved in autophagy regulation, the linkage between the UPR, autophagy, and ATF4/ISR remains unclear (B’chir et al., 2013, Lai and Wong, 2008). Our present study points to SpL modulation by 4HPR as a single stimulus that concurrently activates each of these three arms to restore cellular homeostasis following metabolic perturbations, such as those occurring during culture. Together, these function as a set of coordinated quality control responses, potentially via a cytoprotective mechanism that promotes the persistence of a more regenerative HSC pool following *ex vivo* cytokine activation from quiescence. We speculate that alterations of Cer/DhCer are part of a “lipid biostat” for stress that is in part enacted by the very distinct biophysical properties of Cer and dhCer. As alluded to in yeast and mouse cells (Thibault et al., 2012), altering lipid homeostasis may have major consequences on membrane structure, and this alteration has been proposed to activate the UPR (Contreras et al., 2010, Halbleib et al., 2017). DEGS1 KD was sufficient to activate the ISR arm of the UPR in HSC subpopulations *in vivo* (Figures 5G and 5H). Perhaps UPR sensing of various proteostatic and lipostatic stress is blunted during aging and together with erosion of autophagy flux in aged HSCs responsible for some aging phenotypes. Would SpL modulation be proficient to activate these cellular stress protection programs and rejuvenate HSCs during aging, as suggested for rapamycin (Chen et al., 2009, Luo et al., 2014)?

Our data indicate a need to evaluate potential adverse effects of obesity, which is linked to an increase in Cer levels (Turpin et al., 2014), on SpL homeostasis in human hematopoiesis. Obesity is also linked to disruption of the murine bone marrow hematopoietic niche and altering HSC function, which persists in cells transplanted from obese mice into a normal environment (Naveiras et al., 2009, Zhou et al., 2017, Lee et al., 2018). We wonder whether obesity reprograms HSC stress responses via SpL lipostatic stress. Moreover, SpL dysregulation is associated with incomplete autophagy in a number of human neuropathies resulting from germline mutations in SpL genes, which manifest as lysosomal lipid storage disorders (Hannun and Obeid, 2018). Interestingly, SpL gene expression was higher in neural stem cells than activated progeny, and the lysosome, which serves as a major site for SpL degradation, was proposed to be critical for quality control of neural stem cells during aging (Leeman et al., 2018). Determining the coordination of SpL metabolism, autophagy regulation, and lysosomal biogenesis may prove informative for understanding how adult stem cells maintain life-long self-renewal. These data predict that SpL metabolism regulates adult stem function in both blood and brain and perturbation of lipostatic stress sensing in the lysosome or other organelles may contribute to human disease and aging.

The study of HSC stress responses has implications beyond understanding stemness control. The ability to control the cellular properties of human HSCs has important clinical implications, particularly for HSCT. Although 50,000 HSCT procedures occur annually, two-thirds of patients who need HSCT lack matched donor tissue (Kiernan et al., 2017, Lund et al., 2015). Limitations of CB as a source for HSCT include the delay in neutrophil recovery following transplantation and long-term sustainability of the donor graft (Lund et al., 2015). We found that DEGS1 inhibition regulates the erythroid-myeloid axis *in vitro*, where there was no alteration of S1P levels, suggesting such lineage commitment changes resulted from dysregulation of SpL composition, not S1P signaling. Although we focused on a cell-autonomous aspect of this biosynthetic pathway, our results are also consistent with recent work in the field on the role of S1P signaling axes on lymphoid and erythroid/platelet lineage commitment (Blaho et al., 2015, Vu et al., 2017). We observed a decrease in S1P levels in shDEGS1 cells isolated from the xenograft where there was also disruption of B lymphoid engraftment. Intrinsic genetic perturbation of S1P signaling is sufficient to dysregulate lymphoid commitment and the HSPC hierarchy in mice (Blaho et al., 2015). However, the erythroid-to-myeloid skewing observed with 4HPR treatment could also result from an ER stress-mediated emergency granulopoiesis-like program (Gombart et al., 2007); this lineage bias may aid in faster neutrophil recovery in patients following transplant of an expanded CB. Another major limitation in wider clinical CB HSCT is the small number of HSCs present in a single unit and the inability of many cytokine cocktails to expand LT-HSCs. Our studies highlight the metabolic and proteostatic stress perturbations that arise upon cytokine activation that collectively impair LT-HSC function at the expense of generation and expansion of downstream progeny. SpL modulation by 4HPR during *ex vivo* culture activates cytoprotective proteostasis programs that result in preservation of significantly higher numbers of functional LT-HSCs in the immunophenotypic HSC pool. Notably, we show here that the search for small molecules to increase LT-HSC donor graft sustainability from CB can be achieved with molecules that do not necessarily expand CD34^+^ cells. Thus, *ex vivo* CB expansion regimens, including 4HPR, to activate a lipostatic stress program together with UM171 and SR1 may provide the best compromise between generating large numbers of ST-HSCs and progenitors for rapid engraftment while still sparing functional LT-HSCs to ensure long-term hematopoiesis.

## STAR★Methods

### Key Resources Table

**Table:** 

REAGENT or RESOURCE	SOURCE	IDENTIFIER
**Antibodies**		

FITC–anti-CD45RA	BD	555488
PE–anti-CD90	BD	555596
PECy5–anti-CD49f	BD	551129
V450–anti-CD7	BD	642916
PECy7–anti-CD38	BD	335790
APC–anti-CD10	BD	340923
APCCy7–anti-CD34	BD	custom made by BD
BV711-CD19	BD	563036
Alexafluor700–anti-CD7	BD	561603
Alexafluor700–anti-CD10	BD	563500
BV510-CD45	BD	563891
biotin-Flt3L	BD	N/A
streptavidin-Qdot605	ThermoFisher	Q10101MP
V450-anti-CD15	BD	642917
PE-Cy7-anti-GlyA	Beckman Coulter	PN A71564
PECy5–anti-CD14	Beckman Coulter	PN A22331
FITC-anti-CD3	BD	349201
V500-anti-CD45	BD	560777
PE–anti-CD19	BD	349209
PE–anti-GlyA	Beckman Coulter	A07792
BV786–anti-CD33	BD	740974
PECy5–anti-CD45	Beckman Coulter	A07785
PECy7–anti-CD14	Beckman Coulter	A22331
APC–anti-CD33	BD	551378
APC–anti-CD45	BD	340943
FITC–anti-CD45	BD	347463
V421–anti-CD10	BD	562902
Ki67-PE	BD	556027
Anti-LC3II	NanoTools	5F10
Anti- pEIF2S1	Abcam	ab196460
Goat Anti-mouse secondary antibody Alexa 488	ThermoFisher	A32723

**Biological Samples**		

Human humbilical cord blood samples	Trillium and Credit Valley Hospital	N/A
Mobilized Peripheral Blood	Princess Margaret Cancer Centre	N/A

**Chemicals, Peptides, and Recombinant Proteins**		

DNase I	Roche	11284932001
Ammonium Chloride	Stem Cell Technologies	07850
AmpliTaq Gold 360 Polymerase	ThermoFisher	4398813
BamH1-HF	NEB	R0136S
Mlu1-HF	Invitrogene	15432-016
FLT3 Ligand	Milteny Biotec	130-096-474
IL6	Milteny Biotec	130-093-934
SCF	Milteny Biotec	130-096-696
TPO	Milteny Biotec	130-095-752
EPO	Janssen	Eprex 10,000 IU/ml
IL-3	Milteny Biotec	130-095-068
GM-CSF	Milteny Biotec	130-093-866
Fenretinide/4HPR	Tocris Biosciences	1396
StemRegenin1	Stem Cell Technologies	72344
UM171	Xcessbio	60223-2
DMSO	Fisher Chemical	D128-500
ATRA	Gift from Gordon Keller	N/A
Bafilomycin A1	Sigma	B1793
ISRIB	Selleckchem	S7400
DAPI	Sigma	10236276001

**Critical Commercial Assays**		

StemSep Human Hematopoietic Progenitor Cell Enrichment Kit	Stem Cell Technologies	14066
CD34 MicroBead Kit, human	Milteny Biotec	130-097-047
LS Columns	Milteny Biotec	130-042-401
Mouse Cell Depletion Kit	Milteny Biotec	130-104-694
SuperScript VILO	ThermoFisher	1754050
Roche Lightcycler 480 using Power SYBR Green	ThermoFisher	4367659
LysoTracker blue	ThermoFisher	L7525
CYTO-ID® Autophagy detection kit	Enzo Life Sciences	ENZ-51031-0050
RNeasy Micro Kit	QIAGEN	74004
APC BrdU Flow Kit	BD	552598
CellROX deep red	ThermoFisher	C10422
TMRE	ThermoFisher	T668
RNeasy Plus Micro Kit	QIAGEN	74034

**Deposited Data**		

Index-sorted single cell (sc) RNA-seq data of human HSPCs	Velten et al., 2017	GEO: GSE75478
RNA-seq data	Dick lab, Princess Margaret Cancer Centre	GEO: GSE125214
RNA-seq data for uncultured LT-HSC, ST-HSC and GMP	Dick lab, Princess Margaret Cancer Centre	GEO: GSE125345
Raw RNA-seq data	Dick lab, Princess Margaret Cancer Centre	EGAS00001003756

**Experimental Models: Organisms/Strains**		

NOD.Cg *Prkdc*scid*Il2rg*tm1Wjl /SzJ	The Jackson Laboratory	005557

**Oligonucleotides**		

shRNA amplification Forward primer: GGATCCTGTTTGAATGAGGCTTCAGTACTT TACAGAATCGTTGCCTGCACATCTTGGAAAC ACTTGCTGGGATTACTTCT	Kaufmann et. al, 2019	N/A
shRNA amplification Reverse primer: AGTAAC GCGTAAAGTGATTTAATTTATACCATTTTAATT CAGCTTTGTAAAAATGTATCAAAGAGATAGCAA GGTATTCAGTTTTAGTAAACAAGATAATTGCT CCTAAAGTAGCCCCTTGAAGTCCGAGGCA GTAGGCA	Kaufmann et. al, 2019	N/A
DEGS1 shRNA sequence: TGCTGTTGACA GTGAGCGAGGTCATGAAACTTACTCATAATAGT GAAGCCACAGATGTATTATGAGTAAGTTTCATG ACCCTGCCTACTGCCTCGGA	This paper	N/A
control Renilla shRNA sequence: TGCTGTTGAC AGTGAGCGCAGGAATTATAATGCTTATCTATAGTG AAGCCACAGATGTATAGATAAGCATTATAATTCCT ATGCCTACTGCCTCGGA	This paper	N/A
DEGS1 forward pPCR primer: CAAACATTCCAAACCAGCGAT	This paper	N/A
DEGS1 reverse pPCR primer: GCAGTTGCATTAACCACTCAA	This paper	N/A
GAPDH forward pPCR primer: ACATCGCTCAGACACCATG	This paper	N/A
GAPDH reverse pPCR primer: TGTAGTTGAGGTCAATGAAGGG	This paper	N/A

**Recombinant DNA**		

shRenilla	Kaufmann et. al, 2019	pLBC2 lentiviral vector
shDEGS1	This manuscript	pLBC2 lentiviral vector

**Software and Algorithms**		

FACSDiva	BD	N/A
FlowJo v 9.96	Flowjo, LLC	N/A
Prism 7	Graphpad Software	N/A
Extreme Limiting Dilution Analysis (ELDA)	Hu and Smyth, 2009	http://bioinf.wehi.edu.au/software/elda/
GSEA		http://software.broadinstitute.org/gsea/index.jsp
Pathway database, Bader lab (version April 2017)	http://baderlab.org/GeneSets	N/A
Scran	Lun et al., 2016	https://bioconductor.org/packages/release/bioc/html/scran.html
Scanpy	Wolf et al., 2018	https://github.com/theislab/scanpy
Self Assembling Manifold Algorithm	Tarashansky et al., 2019	https://github.com/atarashansky/self-assembling-manifold

**Other**		

X-Vivo medium	Lonza	04-380Q
Fetal bovine serum	Sigma	F1051-500mL
Iscove’s modified Dulbecco’s medium (IMDM)	GIBCO	12440-053
MethoCult™ Optimum	Stem Cell Technologies	H4034
StemPro™-34 SFM	GIBCO	10640-019
StemPro nutrients	GIBCO	10641-025
L-glutamine	Multicell	609-065-EL
Pen/Strep	GIBCO	15140-122
Human LDL	Stem Cell Technologies	02698

### Lead Contact and Materials Availability

Further information and requests for resources and unique/stable reagents generated in this study should be directed to and are available without restriction from the Lead Contact, John E. Dick (jdick@uhnresearch.ca).

### Method Details

#### Experimental Model and Subject Details

Human cord blood samples were obtained with informed consent from Trillium and Credit Valley Hospital according to procedures approved by the University Health Network (UHN) Research Ethics Board. Mononuclear cells were obtained by centrifugation on Lymphoprep medium (Stem Cell Technologies) and were depleted of Lin+ cells (lineage depletion) by negative selection with the StemSep Human Progenitor Cell Enrichment Kit according to the manufacturer’s protocol (Stem Cell Technologies). Lin^-^ CB cells were stored viably at −80°C or −150°C. Human mobilized peripheral blood samples (mPB) were obtained with informed consent from Princess Margaret Cancer Centre according to procedures approved by the UHN Research Ethics Board. Following mononuclear cell isolation, CD34+ cells from CB and mPB were enriched by positive selection with the CD34 Microbead kit (Miltenyi) and LS column purification with MACS magnet technology (Miltenyi).

#### Cell Sorting

Lin^–^ cells were thawed by dropwise addition of X-VIVO + 50% fetal calf serum supplemented with DNase (100 μg/mL final concentration, Roche) and resuspended at a density of 5 × 10^6^ cells/mL. Cells were then stained with the following antibodies (all from BD, unless stated otherwise): FITC–anti-CD45RA (1:50, 555488), PE–anti-CD90 (1:50, 555596), PECy5–anti-CD49f (1:50, 551129), V450–anti-CD7 (1:33.3, 642916), PECy7–anti-CD38 (1:200, 335790), APC–anti-CD10 (1:50, 340923), APCCy7–anti-CD34 (1:200, custom made by BD). Cells were sorted on FACS Aria III (Becton Dickinson), consistently yielding > 95% purity. LT-HSC were sorted based on the following markers: CD34^+^CD38^-^CD45RA^-^CD90^+^CD49f^+^. ST-HSC were sorted as CD34^+^CD38^-^CD45RA^-^CD90^-^CD49f^-^ and GMP as CD34^+^CD38^+^CD10^-^CD7^-^CD45RA^+^. shCtrl or shDEGS1 previously frozen 4 week xenograft samples were thawed as described above. Mouse cells were depleted with the mouse depletion kit (Miltenyi) and LS column purification with MACS magnet technology (Miltenyi). Cells were then stained with the following primary antibodies: FITC–anti-CD45RA PE–anti-CD90, PECy5–anti-CD49f, BV711-CD19 (1:50, 563036), Alexafluor700–anti-CD7 (1:50, 561603), either APC–anti-CD10 or Alexafluor700–anti-CD10 (1:50, 563500) BV510-CD45 (1:50, 563891), biotin-Flt3L (1:50, 624008), and APCCy7–anti-CD34. Cells were washed and then stained with streptavidin-Qdot605 (1:100, Q10101MP). LT-HSC were sorted based on the following markers: CD45^+^BFP^+^C19^-^CD34^+^CD38^-^CD45RA^-^CD90^+^CD49f^+^. ST-HSC were sorted as CD45^+^BFP^+^CD19^-^CD34^+^CD38^-^CD45RA^-^CD90^-^CD49f^-^ and GMP as CD45^+^BFP^+^CD19^-^CD34^+^CD38^+^CD10^-^CD7^-^ CD45RA^+^.

#### Sphingolipid Quantitation by Mass Spectrometry

To profile the SpL composition of primitive CB cells, 1.05-1.1 million CD34+CD38- and 1.9-2.9 million CD34^+^CD38^+^ cells were isolated from 3 pools of previously frozen lin- CB (22 million – 50 million cells) by flow cytometry, washed with PBS, and frozen as cell pellets. For the SpL distribution of mature CB lineages, freshly isolated CB bags from five individuals were processed for mononuclear cells and a small fraction stained with V450-anti-CD15, APC-cy7-anti-CD34, PE-Cy7-anti-GlyA, PECy5–anti-CD14, FITC-anti-CD3, and V500-anti-CD45 to sort for erythrocytes (GlyA+CD45-) prior to ammonium chloride lysis. After lysis, cells were stained at 10 million/ml and T cells (CD45^+^CD3^+^CD19^-^CD14^-^CD15^-^), B Cells (CD45^+^CD3^-^CD19^+^CD14^-^CD15^-^), monocytes (CD45^+^CD3^-^CD19^-^CD14^+^CD15^-^), and neutrophils (CD45^+^CD3^-^CD19^-^CD14^-^CD15^+^) were isolated by flow cytometry on the Aria Fusion or Aria RITT. Cell numbers ranged from 1-4 million cells. Subsequent lipid extraction and mass spectrometry for sphingomyelin species, hexosylceramide species, ceramide species, dihydroceramide species and sphingoid species were performed by the Lipidomics Facility of Stony Brook University Medical Center (Bielawski et al., 2009). As the morphology between the subpopulations are quite distinct, particularly between the mature lineages, normalization of the sphingolipidome data was an important consideration. Normalization to cellular inorganic phosphate (P_i_) was chosen to minimize the potential confounding effects of differences in cellular size and protein content between the profiled populations. Two pools CD34- lin^-^ CB cultured with DMSO control or 4HPR for 8 days were collected and 4 million expanded cells were washed twice in PBS and frozen as cell pellets. CD45^+^BFP^+^ cells lacking the CD34^+^CD19^-^ population were sorted from shCtrl (1.8-2.7 million cells) or shDEGS1 (0.4-0.5 million cells) previously frozen 4 week xenograft samples (Primary transplant data in Figures S1I–S1O, Experiment 1, n = 2 biological replicates), washed and frozen as cell pellets. Subsequent lipid extraction and mass spectrometry for ceramide species, dihydroceramide species and sphingoid species were performed by the Lipidomics Facility of Stony Brook University Medical Center. Inorganic phosphate levels were measured for lipid normalization across samples.

#### Lentiviral shRNA Knockdown of DEGS1

shRNA sequences were predicted using the Sherwood algorithm (Knott et al., 2014) and ordered as Ultramer DNA oligos (IDT). Subsequently, shRNAs were amplified using AmpliTaq Gold 360 Polymerase (ThermoFisher, 4398813) using FW primer: 5′- GGATCCTGTTTGAATGAGGCTTCAGTACTTTACAGAATCGTTGCCTGCACATCTTGGAAACACTTGCTGGGATTACTTCT-3′ and RV primer: 5′-AGTAACGCGTAAAGTGATTTAATTTATACCATTTTAATTCAGCTTTGTAAAAATGTATCAAAGAGATAGCAAGGTATTCAGTTTTAGTAAACAAGATAATTGCTCCTAAAGTAGCCCCTTGAAGTCCGAGGCAGTAGGCA-3′. The PCR product was digested with BamH1-HF and Mlu1-HF (NEB) and subcloned into the pLBC2 lentiviral vector, downstream of SFFV-tBFP. Viral production, titration and transduction of CD34^+^CD38^-^ CB cells were done as previously described (Kaufmann et al., 2019).

#### shRNA sequences

##### DEGS1 shRNA (shDEGS1)

5′-TGCTGTTGACAGTGAGCGAGGTCATGAAACTTACTCATAATAGTGAAGCCACAGATGTATTATGAGTAAGTTTCATGACCCTGCCTACTGCCTCGGA-3′

##### control Renilla shRNA (shCtrl)

5′-TGCTGTTGACAGTGAGCGCAGGAATTATAATGCTTATCTATAGTGAAGCCACAGATGTATAGATAAGCATTATAATTCCTATGCCTACTGCCTCGGA-3′

#### Quantitative RT-PCR for DEGS1 Knockdown

In order to assess shRNA knock-down efficiency, MOLM13 cells were infected at a multiplicity of infection of 0.3. Transduced cells were sorted for BFP^+^ expression and total RNA was isolated and DNase treated using the RNeasy Micro Kit (QIAGEN, 74004). RNA quality (RIN > 9) was verified using the Bioanalyzer RNA 6000 Pico Kit (Agilent) and cDNA was synthesized using SuperScript VILO (ThermoFisher, 11754050). qPCR was performed on the Roche Lightcycler 480 using Power SYBR Green (ThermoFisher, 4367659). All signals were quantified using the ΔCt method and were normalized to the levels of GAPDH.

#### qPCR primers

##### DEGS1

5′-CAAACATTCCAAACCAGCGAT-3′
5′-GCAGTTGCATTAACCACTCAA-3′

##### GAPDH

5′-ACATCGCTCAGACACCATG-3′
5′-TGTAGTTGAGGTCAATGAAGGG-3′

#### Xenotransplantation

All animal experiments were done in accordance to institutional guidelines approved by the University Health Network Animal care committee. Aged match female or male NSG mice (NOD.Cg *Prkdc*scid*Il2rg*tm1Wjl /SzJ; Jackson Laboratory) 10-12 weeks of age were sublethally irradiated with 250 rads 1 day before intrafemoral injection. Following 4 weeks xenotransplantion of lentiviral DEGS1 knockdown into male NSG, mice were euthanized and the injected femur and other bones were flushed separately in Iscove’s modified Dulbecco’s medium (IMDM) and human chimerism and transduced cells marked by BFP expression were assessed by flow cytometry on the BD Celesta and the following antibodies: PE–anti-CD19 (349209), PE–anti-GlyA (Beckman Coulter, A07792), V500–anti-CD45 (560777), APCCy7–anti-CD34, and BV786–anti-CD33 (740974). For primary transplant analysis of *ex vivo* cultured cells, female mice were euthanized at 16 weeks after transplantation. The injected femur and other bones were flushed separately in Iscove’s modified Dulbecco’s medium (IMDM) and human chimerism was assessed with the following antibodies: PE–anti-CD19 (349209), PE–anti-GlyA (Beckman Coulter, A07792), PECy5–anti-CD45 (Beckman Coulter, A07785), PECy7–anti-CD14 (1:200; Beckman Coulter, A22331), APC–anti-CD33 (551378) and V450–anti-CD15 (642917). For secondary LDA assays, primary transplant mice from each treatment group were individually thawed, and 1/3 of each mouse pooled and stained with APC–anti-CD45 and FITC–anti-CD45. Human CD45+ cells were sorted from each pooled sample and injected at indicated doses into irradiated female NSG mice and engraftment assessed at 16 weeks post-transplant. For LDA experiments, injected femur and non-injected femurs were isolated and flushed separately and analyzed with the following: APC–anti-CD45, FITC–anti-CD45, PE-anti-CD19 and APC–anti-CD33. A mouse was considered engrafted if CD45+ > 0.1 and multilineage. LTRC frequency was estimated using the ELDA software (http://bioinf.wehi.edu.au/software/elda/; Hu and Smyth, 2009). The remaining 2/3 of bone marrow from each individual mouse was first enriched for CD34+ cells by positive selection with the CD34 Microbead kit (Miltenyi) and LS column purification with MACS magnet technology (Miltenyi). CD34+ enriched cells were then directly analyzed by flow cytometry with the following antibodies: FITC–anti-CD45RA, APC–anti-CD90, PECy5–anti-CD49f, V450–anti-CD7, PECy7–anti-CD38, V421–anti-CD10, APCCy7–anti-CD34, PE-anti-CD19, V500-anti-CD45.

#### Methylcellulose CFC Assay

LT-HSC, ST-HSC, or GMP were sorted directly into methylcellulose (cat. No H4034, Stem Cell Technologies), supplemented with FLT3 Ligand (20 ng/ml) and IL6 (50 ng/ml). Fenretinide/4HPR (Tocris Biosciences, cat. # 1396), StemRegenin1 (Stem Cell Technologies, cat. # 72344), and UM171 (Xcessbio, cat. #60223-2), or DMSO vehicle were added following sorting at the following concentrations, DMSO (control), 2 μM 4HPR, 35nM UM171 or 500 nM SR1, such that DMSO was always < 0.1% and equal between treatment and control groups. ATRA was a kind gift from G. Keller. Samples were mixed and plated onto 35 mm dishes in duplicates. Colonies were allowed to differentiate for 10-11 days and morphologically assessed for colonies in a blind fashion by a second investigator. At day 14, colonies from replicate plates were pooled and resuspended in PBS/FBS and stained with FITC–anti-CD45RA, APC–anti-CD90, PECy5–anti-CD14, APCCy7–anti-CD34, PE-anti-CD235a (GlyA) for flow cytometry analysis on a BD Canto or Celesta. For shDEGS1 experiments, sorted LT-HSC, ST-HSC, or GMP were cultured in low cytokine media for 4 hours and then transduced with shCtrl or shDEGS1 lentivirus. At day 3 post-transduction, BFP+ cells were sorted directly into methylcellulose as above for colony scoring 10 days later (day 13 post-transduction). At day 16 post-transduction, colonies were analyzed on the Celesta for BFP and with APC–anti-CD90, PECy5–anti-CD14, APCCy7–anti-CD34, PE-anti-GlyA, and BV786–anti-CD33.

#### *Ex Vivo* Cord Blood Culture Scheme

Lin^-^ CB were thawed via dropwise addition of X-Vivo based thawing media (X-Vivo, 50% FBS+1% DNase), resuspended in cytokine media, viable cells counted, and placed into appropriate cell concentrations for liquid culture (Laurenti et al., 2015): StemPro (Stem Cell Technologies) supplemented with StemPro nutrients (Stem Cell Technologies), L-glutamine (GIBCO), Pen/Strep (GIBCO), human LDL (Stem Cell Technologies, 50 ng/mL) and the following cytokines (all from Miltenyi): SCF (100 ng/mL), Flt3L (20 ng/mL), TPO (100 ng/mL), IL-6 (50 ng/mL), IL-3 (10 ng/mL), GM-CSF (20 ng/mL), except EPO (3 units/mL, from Jansen). The experimental scheme for *ex vivo* culture of viable lin- CB cells post thawing is the following: 3 initial cell doses in a LDA fashion with 62,500 cells (high), 12,500 cells (medium), and 2,500 cells (limiting) are cultured per well in a 24 well plate with 0.5 mls expansion media plus vehicle control or drug(s) on day 0. We mimicked fed-batch growth conditions where drug was added with fresh media every second day for 8 days to reduce auto-inhibitory signaling and ensure nutrients and drugs are not limiting during the culture period (Csaszar et al., 2012): fresh media plus drugs are added on day 2 (0.5 mls), day 4 (1 ml), and feeding and transferring to a 6 well plate on day 6 (2 mls). On day 8, all progeny are collected and 10/11^th^ are unbiasly transplanted via intrafemoral injection into 5 NSG mice/condition. 1 LTRC cell is roughly equivalent to 700 lin- CB cells as previously calculated in xenotransplantation (Notta et al., 2011). This translates to the equivalent of the following day 0 cell doses: ∼11,300 lin- cells or ∼16 initial LTRC/mouse (high), ∼2270 lin- cells or∼3.2 LTRC/mouse (medium) and 454 lin- cells or ∼0.65 LTRC/mouse (limiting). The remaining 1/11^th^ of day 8 progeny are analyzed by flow cytometry on a BD canto with a plate reader to enumerate cell numbers and lineage distribution with the following: V450-anti-CD15, APC-cy7-anti-CD34, PE-Cy7-anti-GlyA, PECy5–anti-CD14, or FITC-anti-CD15, PE-Cy5-anti-CD34, PE-anti-GlyA, PECy7–anti-CD14. LT-HSC, ST-HSC and GMP were sorted and ∼500 cells/well were cultured in 100 μL of cytokine media with DMSO on a 96 well plate on day 0. On day 4, an additional 100 μL of *ex vivo* media with compounds were added. Cells were stained with APCCy7–anti-CD34 on day 8 on a BD Canto with plate reader. For inhibition of autophagy and the ISR, 20nM Bafilomycin A1 (Sigma, B1793) and 500 nM ISRIB (Selleckchem, S7400), dissolved in DMSO, were added to 20,000 lin- CB cultured in cytokine media as indicated and analyzed for viable cells and lineage as previously and with CytoID (Enzo Life Sciences) or LysoTracker blue (ThermoFisher Scientific, L7525).

#### Proliferation and Cell Cycle Assays

For assessment of proliferation using BrdU incorporation assays, indicated subpopulations were cultured for three days with DMSO vehicle or 2 μM 4HPR in expansion media when BrdU was added to cells for 4 to 8 hours. BrdU staining was performed with the APC BrdU Flow Kit (BD PharMingen) with APC-anti-BrdU according to the manufacturer’s protocol. Sorted LT-HSC, ST-HSC or GMP were cultured in *ex vivo* culture media with vehicle or 4HPR as indicated and fixed and stained for Ki67-Hoechst assays as described (Laurenti et al., 2015). LT-HSC and GMP were sorted from shCtrl or shDEGS1 previously frozen 4 week xenograft samples (Primary transplant data in Figures S1I–S1O, Experiment 1, n = 2 CB) and fixed and stained for Ki67-Hoechst analysis. Samples were analyzed on a BD LSRII cytometer with an UV laser.

#### Immunofluorescence for LC3II And Phosphorylated EIF2α

1x10^4^ sorted CD34+CD38- or CD34+CD38+ cells were cultured in *ex vivo* culture media on a 96 well suspension plate with DMSO or 2 μM 4HPR. Bafilomycin A1 was added at 30 hours post-culture. Cells were collected at 48 hours post-culture and fixed with 4% paraformaldehyde. Cells were stain with a mouse monoclonal antibody to LC3II (NanoTools, 5F10) and DAPI. Slides were visualized on a Zeiss LS700 confocal microscope and images collected. Quantitation of LC3II foci area as a percentage of total cell area was done using R and ImageJ software (n = 3 CB). A range of 50-150 cells were quantified per condition. LT-HSC and GMP were purified sorted from CD34^+^ CB or mPB samples, fixed with 4% paraformaldehyde and stained with LC3 and DAPI as above. Following image collection on a Zeiss LS700 confocal microscope, LC3II integrated density (total signal intensity) was quantified using R and ImageJ software (n = 3). LT-HSC and ST-HSC were sorted from shCtrl or shDEGS1 previously frozen 4 week xenografts samples (n = 3, Experiment 2, Figures S1I–S1O), fixed with 4% paraformaldehyde and stained with a rabbit monoclonal antibody to EIF2S1 (phospho S51, Abcam, ab196460) and DAPI and images collected as above.

#### CytoID, ROS, Mitochondria Membrane Potential Analysis

1x10^4^ sorted CD34^+^CD38^-^ or CD34^+^CD38^+^ cells were cultured in *ex vivo* media on a 96 well suspension plate with DMSO, 0.2 μM or 2 μM 4HPR for either 2 days or 3 days. Autophagy flux was measured using CytoID analysis kit (Enzo Life Sciences). Cytokine withdrawal experiments to induce autophagy were done by replacing media containing only drugs without cytokines for 6 hours on cells and then analyzing with CytoID. Staining for ROS and mitochondrial membrane potential in active mitochondria was performed by incubating cells at 37°C with 5 μM CellROX deep red (C10422) and 1 μM TMRE (T668), following the manufacturer’s protocols (ThermoFisher) and directly analyzed on a BD celesta.

#### Rna-Sequencing and Pathway Analysis

Three pools of lin- CB at the 16 LTRC dose (62,500 cells/well, 24 well plate) were cultured with *ex vivo* culture media and the following 4 treatment conditions: DMSO (control), 2 μM 4HPR, 35nM UM171 + 500 nM SR1 (U+S) or combination of 4HPR+UM171+SR1 (3-Factor). Cultured cells at day 2 and day 4 were collected, pelleted, washed twice with PBS and resuspended in RNeasy microRNA plus kit (QIAGEN) and frozen for subsequent RNA isolation. Sufficient cells were cultured to isolate a minimum of 500 ng RNA. Nextera libraries were generated without amplification and subjected to 125 bp, paired-end RNA-sequencing on the Illumina HiSeq 2500 with an average of ∼57 million reads/sample at the Center for Applied Genomics, Sick Kids Hospital. Reads were trimmed to remove the adapters and were then aligned to hg19 using TopHat (v2.1.1) and gene counts were generated using HTSeq (v 0.6.1). Read counts were retrieved for each sample and processed using edgeR to estimate differential expression between the treated and ctrl samples and between 3-Factor and U+S and 4HPR. Genes with count per million equal or less than 0.25 in at least one fifth of the samples were removed from further analysis. 15085 genes remained in the analysis. edgeR dispersion parameters were estimated for the whole dataset and a generalized model was applied to compare each treated-control pair. Cord blood batch was included in the model. Tests were corrected for multiple hypothesis testing using the Benjamini-Hochberg method. MDS plots, hierarchical clustering and heatmaps were generated using logarithm of base2 of count per million of TMM normalized counts. A score to rank genes from top upregulated to downregulated was calculated using the formula -sign(logFC) ^∗^ -log10(pvalue). The rank file from each comparison was used in pathway analysis (GSEA) (http://software.broadinstitute.org/gsea/index.jsp) using 2000 permutations and default parameters against a pathway database containing Msigdb c2 and c3, NCI, IOB, NetPath, HumanCyc, GO BP and Panther (http://baderlab.org/GeneSets, version April 2017). NES and FDR results are located in Table S1. EnrichmentMap version 2.1.0 in Cytoscape 3.4.0 was used to visualize enriched pathway gene sets at FDR ≤ 0.05 with a Jaccard coefficient set to 0.25. For GSEA analysis in Figure 5B and Velten et al. scRNA-seq, lists of genes were returned by taking the union of all gene sets for the pathways in Figure 5A.

#### Single Cell Rna-Seq Analysis

Index-sorted single cell (sc) RNA-seq data of human HSPCs (Velten et al., 2017) were obtained from GEO (GSE75478). Raw count data and FACS surface marker annotations were loaded into the Scanpy single cell analysis suite (Wolf et al., 2018). FACS surface marker measurements were subject to logicle transformation through the flowutils package (https://github.com/whitews/FlowUtils), and used to distinguish CD34^+^CD38^-^ plates from CD34^+^CD38^+^ plates for each individual. Among the CD34^+^CD38^-^ plates, CD34^+^CD38^-^CD45RA^-^ HSPCs were identified and their gene expression counts were subjected to pooling normalization using the scran package in R (Lun et al., 2016). Normalized CD34^+^CD38^-^CD45RA^-^ HSPCs for each individual were subsequently clustered using the ‘Self Assembling Manifolds’ (SAM) algorithm, which iteratively re-scales gene expression to identify subpopulations with subtle, yet consistent, differences (Tarashansky et al., 2019). SAM returned two distinct clusters for each individual, with one cluster demarcated by higher CDK6 expression, expression of cell cycle genes (Tirosh et al., 2016), and surface CD38 expression as measured through FACS. Cells in the second cluster expressed fewer distinct genes but displayed higher relative expression of genes associated with HSC dormancy from murine studies (Cabezas-Wallscheid et al., 2017, using genes with logFC > 2 and FDR < 0.01 in dHSC versus aHSC and MPP). We thus labeled the clusters ‘cell cycle-primed’ and ‘non-primed’, respectively. Among CD34^+^38^−^45RA^-^ cells from both individuals, we confirmed that the cell cycle-primed cluster had significantly higher expression of DEGS1 and of genes associated with ER Stress/UPR and protein folding, but comparable expression for genes associated with ROS and autophagy, consistent with our results for LT-HSCs. Signature scores for gene expression programs were derived by comparing the relative expression of that gene set with a random set of genes in the transcriptome (Tirosh et al., 2016) as implemented in Scanpy. LT-HSC were represented in both the cell cycle-primed cluster and the non-primed cluster. To examine LT-HSC specifically, we identified CD34^+^38^−^45RA^-^ cells that were within the top 40^th^ percentile for both CD49f and CD90 surface marker expression.

#### Statistical Analyses

GraphPad Prism was used for all statistical analyses except RNA-seq. Unless otherwise indicated, mean ± SD values are reported in the graphs. Statistical significance was determined with Student t tests. p < 0.05 (^∗^), p < 0.01 (^∗∗^), p < 0.001(^∗∗∗^), and p < 0.0001(^∗∗∗∗^).

### Data and Code Availability

The accession number for the 4HPR RNA-seq data in this paper is GEO: GSE125214 and the accession number for uncultured LT-HSC, ST-HC, and GMP RNA-seq data is GEO: GSE125345. Raw data are available on EGA under accession number EGAS00001003756.

## Supplemental Information

Document S1. Figures S1–S7 and Table S3

Table S1. Table of GSEA Pathway Analysis for RNA-Seq Data from *Ex Vivo* Treated CB Cells, Related to Figures 5 and S5

Table S2. Gene Lists for Selected 4HPR-Altered Pathways, Related to Figures 5 and S5

Document S2. Article plus Supplemental Information
