# Supplementary material for: Sphingolipid Modulation Activates Proteostasis Programs to Govern Human Hematopoietic Stem Cell Self-Renewal
Source: Cell Stem Cell. 2019 Nov 7;25(5):639–653.e7. doi: 10.1016/j.stem.2019.09.008 (PMC6838675; doi:10.1016/j.stem.2019.09.008)
Supplement: Document S1. Figures S1–S7 and Table S3 [file mmc1.pdf]

Cell Stem Cell, Volume *25*

Supplemental Information

Sphingolipid Modulation Activates Proteostasis Programs to Govern Human Hematopoietic Stem Cell Self-Renewal

Stephanie Z. Xie, Laura Garcia-Prat, Veronique Voisin, Robin Ferrari, Olga I. Gan, Elvin Wagenblast, Kerstin B. Kaufmann, Andy G.X. Zeng, Shin-ichiro Takayanagi, Ishita Patel, Esther K. Lee, Joseph Jargstorf, Gareth Holmes, Guy Romm, Kristele Pan, Michelle Shoong, Aditi Vedi, Chiara Luberto, Mark D. Minden, Gary D. Bader, Elisa Laurenti, and John E. Dick

Figure S1, related to Figure 1

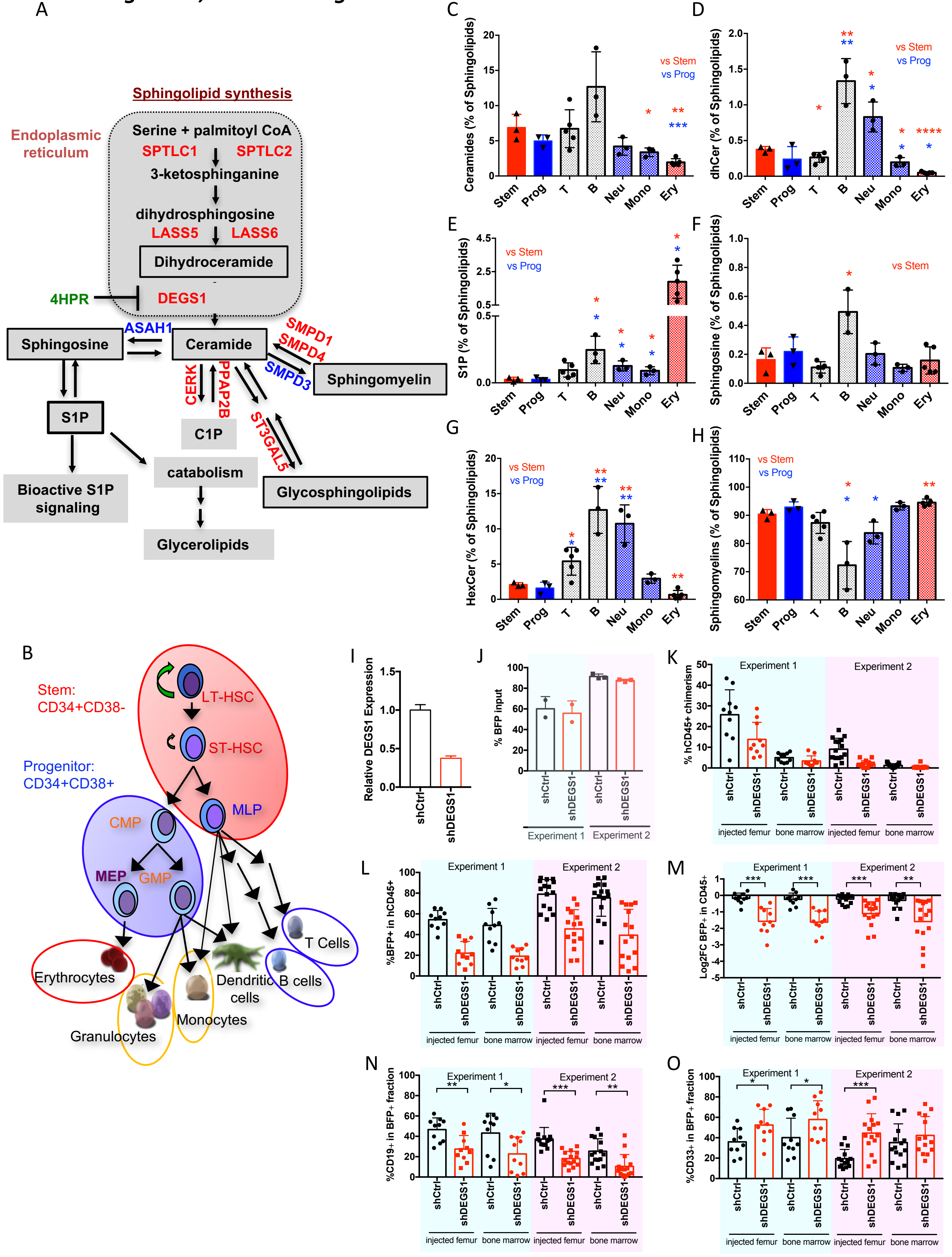

Figure S1, related to Figure 1. DEGS1 contributes to the distinct wiring of sphingolipid synthesis in the human hematopoietic hierarchy and is functionally required *in vivo*. (A) Schematic showing where the SpL genes from Figure 1A lie in the SpL metabolic pathway. Genes more highly expressed in LT-HSC/ST-HSC are in red and genes more highly expressed in committed progenitors are in blue. DEGS1 is a biosynthetic enzyme in *de novo* SpL synthesis, which occurs in the ER, and its activity can be inhibited by 4HPR. (B) Schematic showing the human hematopoietic hierarchy and the 2 HSPC populations and 5 mature populations isolated from CB for measurement of the SpL species indicated in Figure S1A in black boxes by LC/MS. (C) Cer, (D) dhCer, (E) S1P, (F) sphingosine (Sph), (G) hexCer, and (H) SM as percentage of all SpLs measured. (I) KD efficiency of shDEGS1 was quantified in MOLM13 with shCtrl or shDEGS normalized to GAPDH expression. Two separate lentiviral KD experiments in CB $CD34^{+}CD38^{-}$ cells were performed and presented separately (J-O). (J) The transduction input is the % of BFP^{+} cells at 3 days post-transduction for Experiment 1 (n=2 CB pools) and Experiment 2 (n=3 CB pools). Each CB pool was transplanted into 5 individual mice and (K) human $CD45^{+}$ chimerism, (L) % BFP^{+} in $CD45^{+}$ cells, and (M) log2 fold change of BFP^{+} cells ($CD45^{+}$-output vs input), (N) % $CD19^{+}$ cells in BFP^{+} fraction and (O) % $CD33^{+}$ myeloid cells in the BFP^{+} fraction at 4 weeks post-transduction in the injected femur and contra-lateral bones of each mouse was measured by flow cytometry.

Figure S2, related to Figure 2

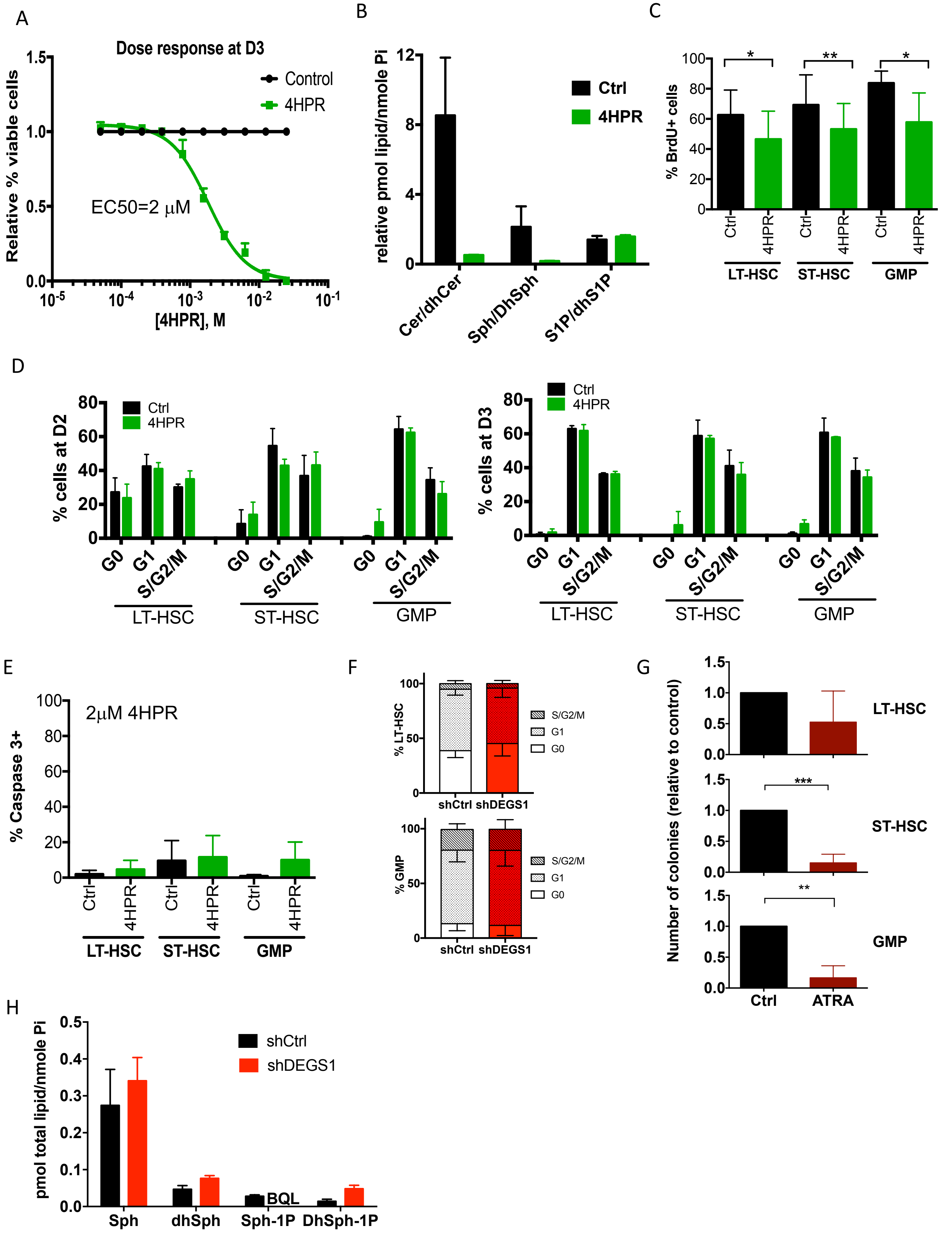

Figure S2, related to Figure 2. Sphingolipid modulation of DEGS1 alters HSC function and lineage balance *in vitro*. (A) Dose response of varying [4HPR] in lin- CB at day 3 post-treatment showing 2uM 4HPR gives the relative half maximal number of viable cells compared to control (n=3). (B) Relative levels of Cer/dhCer, sphingosine (sph)/dihydrosphingosine (dhSph), and S1P/dihydro-S1P (dhS1P) levels in lin- CB cells cultured for 8 days with control or 4HPR (n=2) as measure by lipid LC/MS. The relative levels of lipid/dihydro-lipid show that 4HPR causes accumulation of dhCer and decreases total Cer. However, relative levels of the signaling lipid S1P compared to dhS1P is unchanged by 4HPR treatment compared to control. (C) % BrdU+ cells following 4 or 8 hours labeling at 3d post-treatment with the indicated sorted cell populations shows 4HPR decreases the proliferation rate of primitive CB subpopulations in culture (n=4, paired t-Test). Cell cycle analysis for Ki67 and DNA content by flow cytometry of sorted LT-, ST-HSC, or GMP at (D) day 2 (n=3) or day 3 post treatment (n=2) with vehicle control or 4HPR shows 4HPR does not significantly prevent sorted LT-HSC, ST-HSC or GMP grown in 4HPR from exiting quiescence and transiting through the cell cycle. (E) Flow cytometry for % of cells exhibiting cleaved Caspase 3 with 2uM 4HPR treatment (n=4) at day 3. (F) LT-HSC or GMP purified from 4 week xenografts expressing shCtrl or shDEGS1 by flow cytometry were analyzed for cell cycle distribution with Ki67 and DNA content. (G) Relative colonies formed by LT-HSC, ST-HSC, or GMP treated with $2\ \mu M$ ATRA in CFC assays following 10 days. (H) LC/MS analysis for Sph, dihydrosphingosine (dhSph), Sph-1P and dhSph-1P. Species below quantitation level (BQL) are indicated.

Figure S3, related to Figure 3

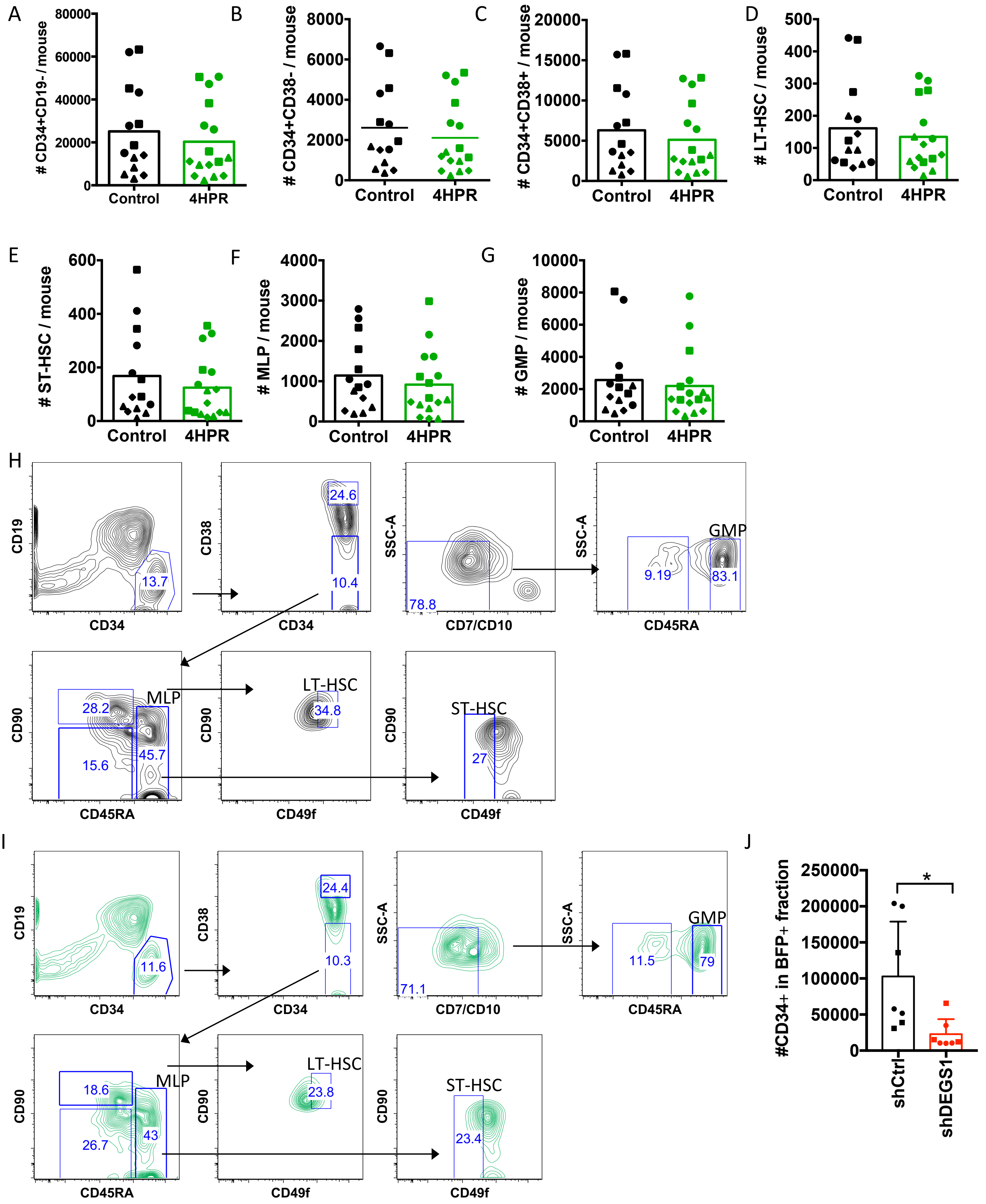

Figure S3, related to Figure 3. ***Ex vivo*** **treatment with 4HPR maintains HSC function following xenotransplantation**. (A-I) Flow cytometry analysis were performed on human CD34+ enriched cells following Miltenyi human CD34 enrichment from mice engrafted with control or 4HPR-treated cells at the 16 HSC dose for the human CB hierarchy from 4 biological experiments, marked with different symbols (n=4 mice per biological experiment). The number of (A) CD34+CD19- cells, (B) CD34+CD38-, (C) CD34+CD38+, (D) LT-HSC, (E) ST-HSC, (F) MLP, and (G) GMP were quantitated from each mouse at 16 weeks post-transplant. Representative HSC hierarchy analysis scheme of CD34 enriched cells isolated from mice 16 weeks post-transplantation engrafted with *ex vivo* cultured CB cells in the presence of (H) vehicle control (black) or (I) 4HPR (green). (J) Mice from Figure 1F-G were pooled and analyzed for the number of CD34+ shCtrl or shDEGS1 BFP+ transduced cells in the CD45+ fraction (mice were pooled for CB xenografts, technical duplicates for 2 CB xenografts).

Figure S4, related to Figure 4

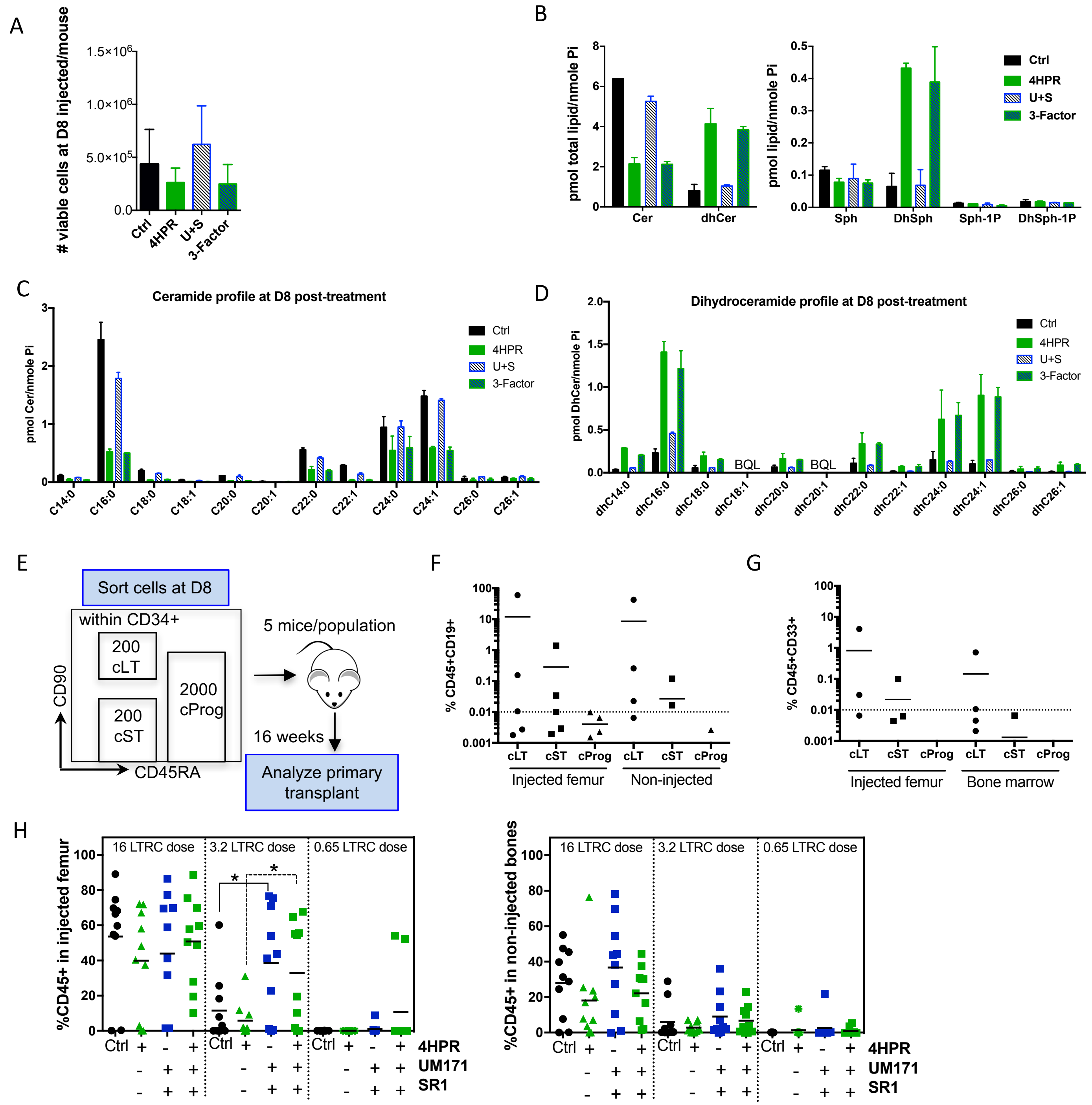

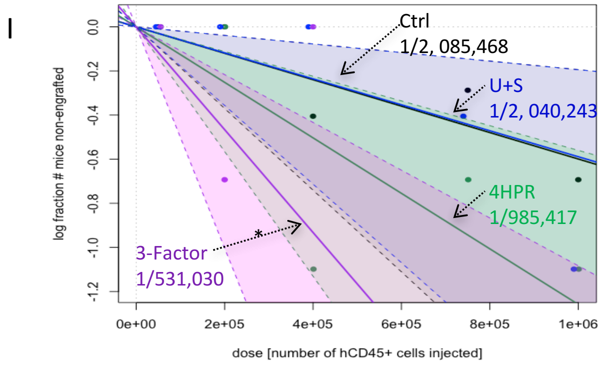

J

Drug treatment	Cell Dose	Tested	Engrafted	LTRC frequency	p value	Fold Change
Control	1000000	2	1	1/2,085,468	-	-
	750000	4	1			
	400000	3	1			
	200000	4	0			
	50000	8	0			
4HPR	1000000	3	2	1/985,417	0.280	2.11
	750000	4	2			
	400000	3	2			
	200000	4	0			
	50000	7	0			
U+S	1000000	3	2	1/2,040,243	0.979	1.02
	750000	3	1			
	400000	3	0			
	200000	4	0			
	50000	7	0			
3-Factor	1000000	3	3	1/531,030	0.043	3.92
	750000	3	3			
	400000	3	0			
	200000	2	1			
	50000	6	0			

Figure S4, related to Figure 4. Sphingolipid modulation with 4HPR restricts expansion of committed progenitors during *ex vivo* culture to enhance HSC self-renewal. (A) The number of cells following 8 days of ex vivo culture at the 16 LTRC dose transplanted per mouse at the indicated drug treatments (n=2). (B) LC/MS analysis for total Cer, dhCer, Sph, dhSph, Sph-1P and dhSph-1P levels show that 4HPR increases dhCer and dhSph alone or in combination with UM171 and SR1 (n=2). (C) The Cer and (D) dhCer profiles normalized to cellular inorganic phosphate levels with the indicated fatty-acyl chain of cells collected following 8 days of culture with indicated treatments. Species below quantitation level (BQL) are indicated. (E) The indicated cell populations were sorted from the CD34+ fraction of lin-CB cultured for 8 days and transplanted into NSG mice to determine human lymphoid and myeloid engraftment. (F) Lymphoid (CD45+CD19+) and (G) myeloid (CD45+CD33+) engraftment from S5A at 16 weeks post-transplantion, Engraftment considered positive at 0.01%. (H) Human CD45 chimerism in the injected femurs and non-injected bones of transplanted mice for the indicated cell doses at 16 weeks xenotransplantion following 8 days *ex vivo* culture with indicated drugs. (I-J) LTRC frequency plot and table from serial transplantation for 2 biological experiments with control, 4HPR, U+S, and 3-Factor treatments were calculated separated and shown.

Figure S5, related to Figure 5 and Table S1,S2 and S3

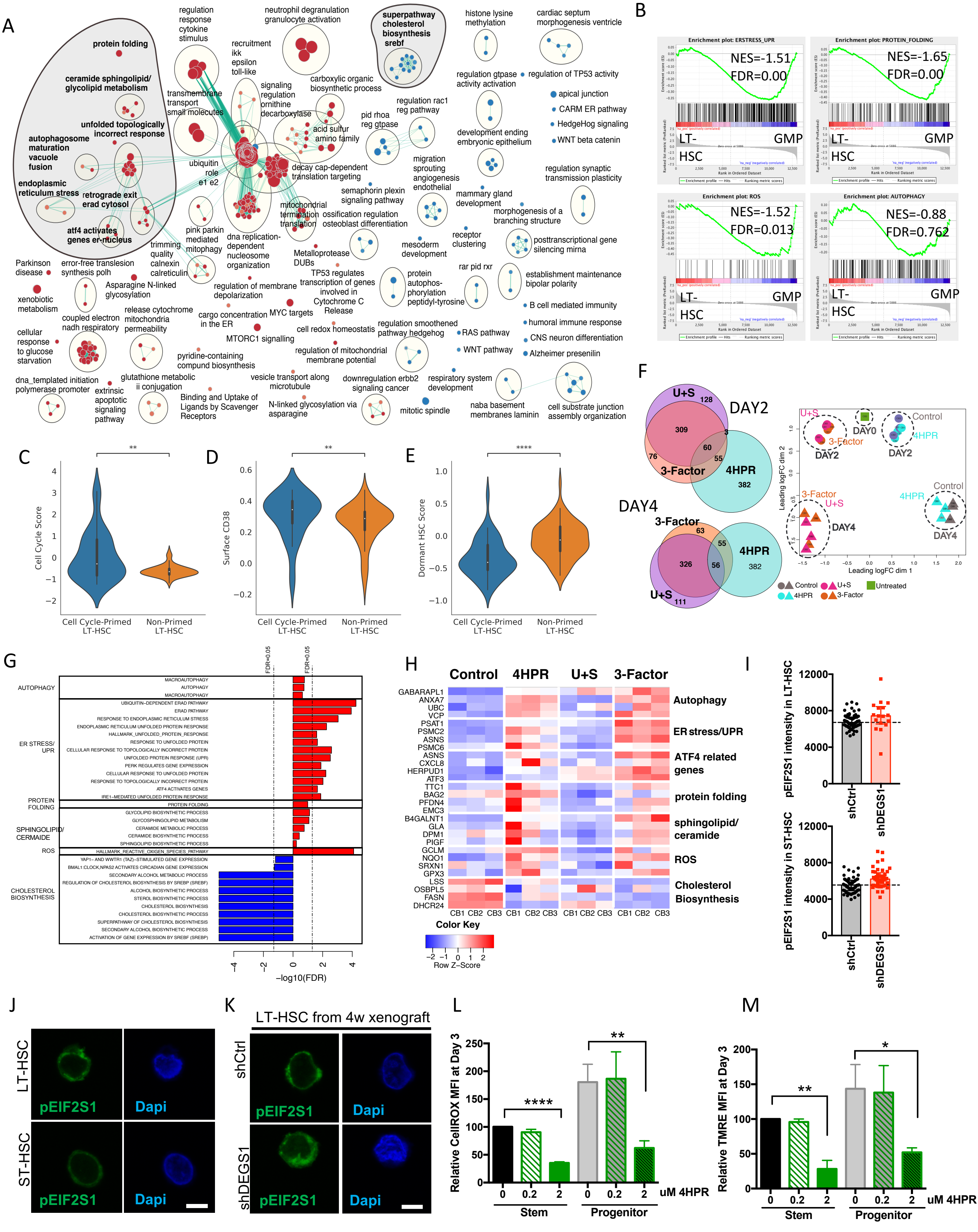

Figure S5, related to Figure 5 and Tables S1, S2 and S3. Sphingolipid modulation with 4HPR treatment upregulates cellular stress pathways and remodels cellular metabolism in HSPC during *ex vivo* culture. (A) Enrichment map at day 2 of 4HPR gene-sets with positive and negative normalized enrichment score (NES) at FDR<0.05 relative to control treatment. Node size is proportional to NES. Red node: positive NES and enrichment in genes up-regulated by 4HPR treatment. Blue node: negative NES and enrichment in genes down-regulated by 4HPR treatment. Green edges indicate gene overlap. (B) GSEA of autophagy, ERstress/UPR, protein folding or ROS pathways in uncultured LT-HSC vs GMP. (C-E) LT-HSC from Velten, et al. were clustered as cell cycle-primed or non-primed as described in methods: (C) cell cycle programs, (D) CD38 surface expression, and (E) dormant HSC programs are shown. significance calculated with Wilcoxon rank sum test for S5C-E, p<0.05 (*), p<0.01 (**), p<0.001(***), p<0.0001 (****). (F) Venn-Diagram at day 2 and day 4 showing number of overlap between top 500 up regulated genes in 4HPR, U+S and 3-Factor treatments. Multi Dimension Scaling (MDS) plot (right) showing similarities between samples at day 2 and day 4. Samples with closest distances are circled with dashed lines. (G) NES scores at day 4 for indicated gene sets from Figure 5A with 4HPR treatment relative to control. (H) Heatmap of gene expression at day 4 for the top 4 genes in selected functional modules from Figure 5B upregulated by 4HPR treatment at day 2. (I) pEIF2S1 intensity for individual cells from 1 CB for BFP^{+} isolated from 4 week xenografts engrafted with shCtrl or shDEGS1 stem cells. Representative pEIF2S1 confocal microscopy images for (J) shCTRL LT-HSC and shCtrl ST-HSC and (K) shCtrl or shDEGS1 LT-HSC. Flow cytometry analysis for (L) relative CellROX and (M) relative mitochondrial membrane potential with TMRE in the progeny of CD34+CD38- stem or CD34+CD38+ progenitor CB cells at day 2 post-treatment with indicated concentrations of 4HPR.

Figure S6, related to Figure 6

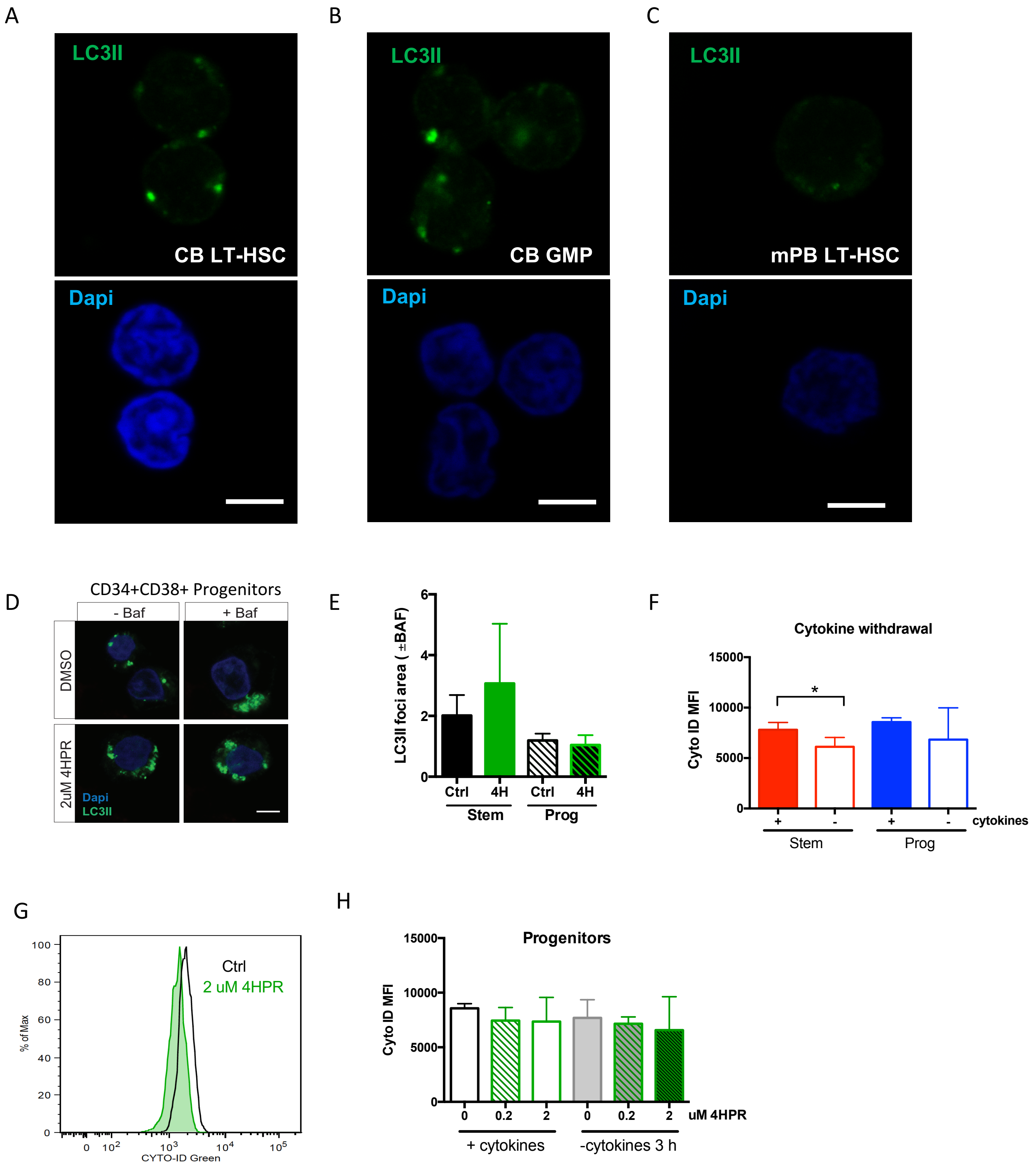

Figure S6, related Figure 6. 4HPR activates autophagy in HSPC during *ex vivo* culture. Representative immunofluorescence images of DAPI (blue) and LC3II staining (green) for uncultured (A) CB LT-HSC, (B) CB GMP, (C) mPB LT-HSC, and (D) CD34+CD38+ cells (prog) following 2 days of treatment with control or 2 μM 4HPR with and without BAF for 12 hours. Scale is 5 μm. (E) Autophagic flux quantification as a ratio of LC3II foci area with and without BAF for Figure 6D-E (n=3). (F) Cyto ID MFI following 3 hours of cytokine withdrawal at 2 days post in vitro culture shows only stem, but not progenitor cells activate autophagic flux upon cytokine withdrawal. (G) Representative flow cytometry histogram plot of Cyto-ID fluorescence intensity of control (black) and 4HPR (green) treated stem cells to assay for autophagic flux. Lower mean fluorescence intensity (MFI) indicates more turnover of autophagosomes and thus increased autophagic flux. (H) CytoID MFI measurements for Figure 3G and the comparable data for progenitor samples to illustrate autophagic flux is not significantly induced in progenitor cells treated with 4HPR even upon cytokine withdrawal.

Figure S7, related to Figure 7

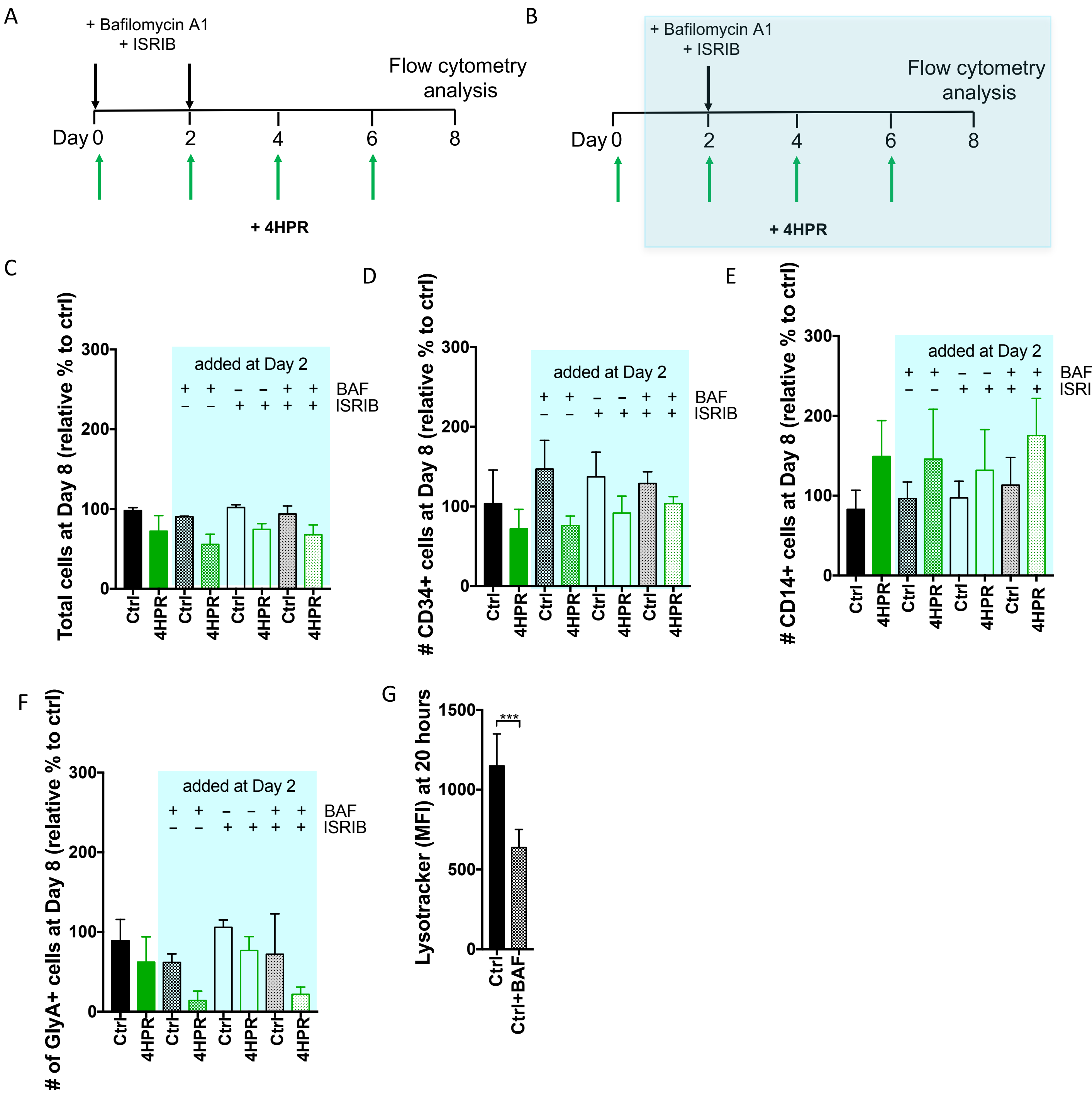

Figure S7, related to Figure 7. Sphingolipid modulation by 4HPR activates a coordinated proteostatic pro-survival response by autophagy and the Integrate Stress Response pathways. (A) Experimental scheme for Figure7A-D. (B) Experimental scheme for Figure S7C-F where BAF and ISRIB are added at day 2 only. Flow cytometry analysis at 8 days post culture for (C) total number of live cells (D) number of CD34+ cells (E) number of CD14+ cells and (F) number of GlyA+ cells represented as the relative % to ctrl treatment. (F) Lysotracker MFI was quantified by flow cytometry in DMSO control treated cells with and without BAF at 20 hours post-treatment.

Table S1, related to Figure 5 and S5. Table of GSEA pathway analysis for RNAseq data from *ex vivo* treated CB cells.

Table S2, related to Figure 5 and S5. Gene lists for selected 4HPR-altered pathways.

Table S3, related to Figure 5 and S5. Table of top 10 differentially expressed genes between Control and 4HPR treatment at day 2 for indicated pathways of Figure 5F.

Table S3

Autophagy							
gene_name	description	logFC	logCPM	LR	PValue	FDR	score
GABARAPL1	GABA type A receptor associated protein like 1	1.823	3.960	23.923	1.00E-06	1.89E-03	5.999
ANXA7	annexin A7	0.492	7.366	16.602	4.61E-05	0.037	4.336
UBC	ubiquitin C	0.677	9.738	15.045	1.05E-04	0.072	3.979
VCP	valosin containing protein	0.476	9.164	11.182	8.26E-04	0.328	3.083
SQSTM1	sequestosome 1	0.648	7.113	8.984	0.0027	0.721	2.565
RHEB	Ras homolog, mTORC1 binding	0.276	6.526	3.438	0.064	1.000	1.196
RRAGB	Ras related GTP binding B	0.325	2.785	2.587	0.108	1.000	0.968
FAM134B	NA	0.441	2.552	2.539	0.111	1.000	0.954
VPS37A	VPS37A, ESCRT-I subunit	0.254	4.867	2.500	0.114	1.000	0.944
ATG4A	autophagy related 4A cysteine peptidase	0.276	3.837	2.488	0.115	1.000	0.941
**ER stress/UPR**							
gene_name	description	logFC	logCPM	LR	PValue	FDR	score
PSAT1	phosphoserine aminotransferase 1	1.142	7.062	30.026	4.26E-08	1.61E-04	7.370
PSMC2	proteasome 26S subunit, ATPase 2	0.646	7.574	27.315	1.73E-07	4.35E-04	6.762
ASNS	asparagine synthetase (glutamine-hydrolyzing)	0.721	6.225	19.579	9.65E-06	0.0108	5.015
PSMC6	proteasome 26S subunit, ATPase 6	0.577	7.162	14.872	1.15E-04	0.0754	3.939
HSPA5	heat shock protein family A (Hsp70) member 5	0.458	10.488	14.327	1.54E-04	0.0918	3.814
PSMC1	proteasome 26S subunit, ATPase 1	0.623	8.108	11.212	8.13E-04	0.3279	3.090
VCP	valosin containing protein	0.476	9.164	11.182	8.26E-04	0.3279	3.083
HSP90B1	heat shock protein 90 beta family member 1	0.481	10.267	10.401	1.26E-03	0.4418	2.900
UBXN4	UBX domain protein 4	0.414	7.567	8.386	3.78E-03	0.8744	2.422
CXCL8	C-X-C motif chemokine ligand 8	2.129	5.389	7.826	5.15E-03	1.0000	2.288
**Protein folding**							
gene_name	description	logFC	logCPM	LR	PValue	FDR	score
UBC	ubiquitin C	0.677	9.738	15.045	1.05E-04	0.072	3.979
HSPA5	heat shock protein family A (Hsp70) member 5	0.458	10.488	14.327	1.54E-04	0.092	3.814
PSMC1	proteasome 26S subunit, ATPase 1	0.623	8.108	11.212	8.13E-04	0.328	3.090
VCP	valosin containing protein	0.476	9.164	11.182	8.26E-04	0.328	3.083
HSP90B1	heat shock protein 90 beta family member 1	0.481	10.267	10.401	1.26E-03	0.442	2.900
TTC1	tetratricopeptide repeat domain 1	0.311	6.732	4.576	0.032	1.000	1.489
BAG2	BCL2 associated athanogene 2	0.300	5.541	3.663	0.056	1.000	1.255
PFDN4	prefoldin subunit 4	0.404	5.618	2.941	0.086	1.000	1.064
EMC3	ER membrane protein complex subunit 3	0.303	5.778	2.880	0.090	1.000	1.047
ERO1LB	NA	0.376	3.472	2.878	0.090	1.000	1.047

Table S3, cont'd

Sphingolipid/Ceramide							
gene_name	description	logFC	logCPM	LR	PValue	FDR	score
B4GALNT1	beta-1,4-N-acetyl-galactosaminyltransferase 1	3.930	0.725	62.092	3.28E-15	4.94E-11	14.485
GLA	galactosidase alpha	0.444	5.912	6.158	0.013	1.000	1.883
SPTSSA	serine palmitoyltransferase small subunit A	0.152	6.162	1.454	0.228	1.000	0.642
ST3GAL5	ST3 beta-galactoside alpha-2,3-sialyltransferase 5	0.201	2.842	1.341	0.247	1.000	0.608
CLN8	CLN8, transmembrane ER and ERGIC protein	0.244	3.861	1.224	0.269	1.000	0.571
SPTLC3	serine palmitoyltransferase long chain base subunit 3	0.994	-1.547	1.216	0.270	1.000	0.568
LARGE	NA	0.504	-1.114	1.210	0.271	1.000	0.567
SGMS2	sphingomyelin synthase 2	0.321	1.240	0.906	0.341	1.000	0.467
CERS5	ceramide synthase 5	0.178	5.312	0.899	0.343	1.000	0.465
ALDH3A2	aldehyde dehydrogenase 3 family member A2	0.110	5.655	0.877	0.349	1.000	0.457
**ROS**							
gene_name	description	logFC	logCPM	LR	PValue	FDR	score
GCLM	glutamate-cysteine ligase modifier subunit	0.735	5.483	21.018	4.55E-06	0.007	5.342
NQO1	NAD(P)H quinone dehydrogenase 1	0.765	4.523	11.876	0.001	0.286	3.245
SRXN1	sulfiredoxin 1	0.485	5.885	7.564	0.006	1.000	2.225
GPX3	glutathione peroxidase 3	1.593	0.142	4.771	0.029	1.000	1.538
VIMP	NA	0.466	5.269	2.621	0.105	1.000	0.977
GSR	glutathione-disulfide reductase	0.295	7.268	2.528	0.112	1.000	0.951
TXN	thioredoxin	0.337	8.153	2.361	0.124	1.000	0.905
SOD1	superoxide dismutase 1	0.368	8.045	2.243	0.134	1.000	0.872
PRDX1	peroxiredoxin 1	0.293	8.920	1.625	0.202	1.000	0.694
TXNRD1	thioredoxin reductase 1	0.252	8.004	1.422	0.233	1.000	0.632
**Cholesterol Biosynthesis**							
gene_name	description	logFC	logCPM	LR	PValue	FDR	score
DHCR24	24-dehydrocholesterol reductase	-0.474	8.745	14.607	1.32E-04	0.083	-3.878
FASN	fatty acid synthase	-0.583	8.919	4.181	0.041	1.000	-1.389
OSBPL5	oxysterol binding protein like 5	-0.462	3.653	4.120	0.042	1.000	-1.373
LSS	lanosterol synthase	-0.431	6.553	3.092	0.079	1.000	-1.104
TM7SF2	transmembrane 7 superfamily member 2	-0.583	4.323	3.080	0.079	1.000	-1.101
CYP27B1	cytochrome P450 family 27 subfamily B member 1	-0.555	0.603	3.068	0.080	1.000	-1.098
HMGCS1	3-hydroxy-3-methylglutaryl-CoA synthase 1	-0.520	7.935	3.006	0.083	1.000	-1.081
HMGCR	3-hydroxy-3-methylglutaryl-CoA reductase	-0.363	7.682	2.823	0.093	1.000	-1.032
CRTC1	CREB regulated transcription coactivator 1	-0.340	3.368	2.603	0.107	1.000	-0.972
ELOVL6	ELOVL fatty acid elongase 6	-0.306	6.825	2.326	0.127	1.000	-0.896
